# Fc multimers effectively treat murine models of multiple sclerosis

**DOI:** 10.3389/fimmu.2023.1199747

**Published:** 2023-08-11

**Authors:** Jin Wang, Kellie Brown, Caroline Danehy, Emmanuel Mérigeon, Stephen Goralski, Samuel Rice, Kwame Torgbe, Fridtjof Thomas, David Block, Henrik Olsen, Scott E. Strome, Elizabeth A. Fitzpatrick

**Affiliations:** ^1^ Dept. of Microbiology Immunology and Biochemistry, UTHSC, Memphis, TN, United States; ^2^ College of Graduate Health Sciences, UTHSC, Memphis, TN, United States; ^3^ Gliknik, Inc., Baltimore, MD, United States; ^4^ College of Medicine, UTHSC, Memphis, TN, United States; ^5^ Dept. of Pathology, UTHSC, Memphis, TN, United States; ^6^ Div. of Biostatistics, Dept. of Preventive Medicine, UTHSC, Memphis, TN, United States

**Keywords:** autoimmunity, multiple sclerosis, FC, therapeutic, EAE

## Abstract

Multiple Sclerosis (MS) is a chronic neurodegenerative disease with limited therapeutic options. Recombinant Fc multimers (rFc), designed to mirror many of the anti-inflammatory activities of Intravenous Immunoglobulin (IVIG), have been shown to effectively treat numerous immune-mediated diseases in rodents. In this study we used the experimental autoimmune encephalomyelitis (EAE) murine model of MS to test the efficacy of a rFc, M019, that consists of multimers of the Fc portion of IgG2, in inhibiting disease severity. We show that M019 effectively reduced clinical symptoms when given either pre- or post-symptom onset compared to vehicle treated EAE induced mice. M019 was effective in reducing symptoms in both SJL model of relapsing remitting MS as well as the B6 model of chronic disease. M019 binds to FcγR bearing-monocytes both *in vivo* and *in vitro* and prevented immune cell infiltration into the CNS of treated mice. The lack of T cell infiltration into the spinal cord was not due to a decrease in T cell priming; there was an equivalent frequency of Th17 cells in the spleens of M019 and vehicle treated EAE induced mice. Surprisingly, there was an increase in chemokines in the sera but not in the CNS of M019 treated mice compared to vehicle treated animals. We postulate that M019 interacts with a FcγR rich monocyte intermediary to prevent T cell migration into the CNS and demyelination.

## Introduction

Multiple Sclerosis (MS) is a complex immune-mediated neurodegenerative disease characterized by immune cell infiltration of the central nervous system (CNS), demyelinating lesions, and axonal damage leading to progressive paralysis [reviewed in (1)]. The disease course is heterogeneous; most patients exhibit a relapsing-remitting (RRMS) course with no clinical progression between flare-ups (2, 3). Other MS patients develop a progressive form of the disease with either no relapses (primary progressive) or few relapses (secondary progressive) and an accumulation of disabilities from disease onset (3, 4). Currently, there are several immune-modulating therapies for MS e.g. rIFNβ, anti-CD20 (rituximab) and anti- α4β1 antibody (natalizumab), that have been successful in reducing disease severity in RRMS patients. However, they do not cure or prevent disease progression, nor do they work for all MS patients. Collectively, these data highlight the need for additional therapies (5, 6).

Naturally occurring immune complexes (ICs) have immunoregulatory properties in a host of diverse infectious and inflammatory diseases. For instance, chronic viral infection with lymphocytic choriomeningitis virus (LCMV) leads to naturally occurring antigen antibody ICs which inhibit FcγR mediated functions such as antibody dependent cell mediated cytotoxicity (ADCC), antibody dependent cell mediated phagocytosis (ADCP) and antigen presentation (7, 8). Likewise, approximately 5-15% of IVIG is composed of aggregates resulting from idiotype anti-idiotype interactions (9) and clinical studies of Immune thrombocytopenia (ITP) have demonstrated that the anti-inflammatory effects of IVIG directly correlate with the level of immune aggregates in the sera (10). Based on the supposition that it is the Fc portion of the IgG molecule in IVIG that contributes to its anti-inflammatory activity, we generated a series of recombinant proteins consisting of homodimers and highly ordered IgG Fc multimers (11, 12). These Fc multimers bind to FcγRs with high avidity and also inhibit complement. In preclinical models, these Fc multimers effectively treat numerous immune-mediated diseases in rodents such as collagen-induced arthritis, experimental autoimmune neuritis and experimental autoimmune myasthenia gravis (11, 13). Their therapeutic efficacy likely involves multiple mechanisms of action including but not limited to inhibition of ADCC, ADCP, and activation mediated inhibition of the complement cascade. Similar anti-inflammatory activities are also evident in non-human primates, where Fc multimers decrease IL-8 and increase IL-1RA and IL-10 in the sera while reducing expression of CD14 on monocytes (12). These compelling data suggest that Fc multimers may also be an effective therapeutic for MS.

Experimental autoimmune encephalitis (EAE) is a murine model of MS which has been successfully used to test several MS therapeutics including IFNβ and natalizumab (14–16). Pathogenicity in the EAE model is driven by the influx of autoreactive Th1 and Th17 cells into the CNS where they become reactivated and induce an inflammatory cascade that ultimately leads to neurodegeneration [reviewed in (17)]. These Th17 cells are critical to disease pathogenicity and recent studies have demonstrated that the highly pathogenic Th17 cell subset is Th1-like and expresses IL-17, IFNγ and GM-CSF (9, 18–20).

Myeloid cells also contribute to EAE; the number of pro-inflammatory monocytes (Ly6C^hi^ CCR2^+^) increases in the circulation shortly after EAE induction and their depletion with chlodronate liposomes suppresses the development of EAE (10, 21, 22). Similarly, CCR2 KO mice also do not develop EAE, demonstrating the critical role that monocytes play in the disease (23–25). Following migration into the CNS, these cells differentiate into pro-inflammatory macrophages [or myeloid derived DCs (mDCs)] producing inflammatory cytokines such as TNFα, IL-1β, and IL-6 and upregulate genes necessary for antigen presentation to reactivate infiltrating T cells (26). CNS damage is believed to occur through the actions of activated myeloid cells, encephalitogenic T cells, autoantibodies and complement activation and direct cytotoxicity due to T cell activity. The continued destruction of myelin ultimately results in axonal degeneration and progression of clinical symptoms.

In this study we sought to test the hypothesis that Fc multimers have the potential to be developed as effective therapeutics for MS. Using the EAE model, we show that one Fc multimer, M019, effectively treats both early and clinically apparent EAE. These therapeutic effects are associated with reduced spinal cord inflammation and demyelination despite similar numbers of IL-17 and IFNγ positive T cells in the spleens of M019 and vehicle treated EAE mice. Surprisingly, M019 treated mice had significantly higher levels of a defined group of CC and CXC chemokines in their periphery than either vehicle treated EAE mice or untreated controls. Collectively, these data suggest that Fc multimers can effectively treat EAE by preventing the entry of pathogenic cells into the CNS and that these trafficking changes are associated with defined patterns of chemokine elevation in the periphery.

## Materials and methods

### Animals

C57BL/6 or SJL female mice were purchased from Jackson Laboratories (Bar Harbor, ME) at 8-10 weeks of age. All animals were housed in sterile micro-isolator cages on a 12-hour light/dark cycle with sterile food and water ad libitum. Hydrogels were added to the cages twice weekly following development of clinical symptoms. The mice were maintained by the Division of Comparative Medicine at UTHSC according to the guidelines of the Animal Welfare Act.

### EAE induction and therapeutic treatment

C57Bl/6 mice (10/group) were immunized via sc route with 200 µg of myelin oligodendrocyte glycoprotein (MOG_35-55_) emulsified in complete Freunds adjuvant (CFA) (Hooke Laboratories, Lawrence MA). The mice were injected IP with 110-250 ng of pertussis (PTX) at-2 and 24hrs post-immunization. Mice were treated with either M019 or saline (vehicle) via daily sc injections beginning on day 4 post-induction or at onset of symptoms as described in figure legends. The second model tested was the relapsing remitting model of EAE; SJL mice were injected sc with [Ser^140^]-PLP_139-151/_CFA on day 0, followed by IP injection of PTX 2 hrs post-immunization. Mice were treated with increasing doses (0.2mg to 2mg) of M019 via sc injection daily beginning day 4 through day 21 post-immunization. In both models mice were scored and weighed daily using the following scale: 0 = no signs of EAE, 0.5 = distal limp tail, 1=Flaccid tail; 1.5 = Limp tail and hind leg inhibition; 2 = Weakness of both hind limbs; 2.5 = paralysis in one hind limb with partial paralysis in second limb; 3 = paralysis of both hind limbs; 3.5 = paralysis of both hind limbs and inability to right itself; 4= limp tail complete hind limb paralysis and partial front limb paralysis. Mice that reached a score ≥ 4 or ≥30% body weight loss were euthanized.

### Brain and Spinal cord isolation

Mice were euthanized at various timepoints post-immunization and perfused with PBS through the left ventricle to remove blood. The spinal cord and/or brain were harvested. Single cell suspensions of CNS tissue was obtained by digestion with DNAse (50µg/ml) and Collagenase D (1mg/ml) and homogenization at 37°C for 30min using the gentleMACS Octo Dissociator with heaters (Miltenyi Biotec, Auburn, CA). The cells were filtered through a 70µM filter, centrifuged and the myelin debris removed by resuspending the pellet in 37% percoll and centrifuging at 860g for 20 min. The cell pellet was resuspended in culture media and used for cell surface flow cytometry and intracellular cytokine staining.

### Flow cytometry, intracellular cytokine staining and M019 binding

Cell surface flow cytometry was performed on isolated spinal cord or spleen cells using the following fluorochrome-conjugated antibodies:CD11b (clone M1/700), CD45 (clone 30-F11), Ly6C (clone HK1.4), CD115 (clone AFS98), SIGLEC H (clone 551), CX3CR1 (clone SAO11F11), F4/80 (clone BM8), NK1.1 (clone PK136), CD80 (clone 16-10A0), IA/IE (clone M5/114.15.2), CD19 (clone ebio1D3), CD4 (clone RM4-5), CD8 (clone 53-6.7), βTcR (clone H57-597), CD44 (clone IM7) and CD69 (clone H1.2F3) (Biolegend, CA or eBioscience, San Diego, CA). A minimum of 10,000 events/sample were collected on a Bio-Rad ZE5 Cell Analyzer (Bio-Rad, CA). Expression of cell surface markers were analyzed using FlowJo software version10.7.2 (BD Biosciences).

For intracellular cytokine staining, spleen cells were incubated overnight with plate bound anti-CD3 and soluble anti-CD28 antibodies in the presence of protein transport inhibitor cocktail (eBioscience) for the last 4hrs of culture. The cells were harvested and incubated with antibodies to CD4 and CD8 and then fixed and permeabilized with 1% saponin followed by incubation with IFNγ (Clone XMG1.2) or IL-17 (clone eBio1787) antibodies. To measure Treg levels, cells were harvested from spleen and incubated with antibodies to CD4, TcRβ and CD25 followed by fixation and permeabilization (eBioscience) and incubation with FoxP3 (clone FJK-16s) antibody based on manufacturer’s instructions. A minimum of 10,000 events/sample were collected on the Bio-Rad ZE5 Cell analyzer and expression of intracellular cytokines was analyzed using FlowJo software.

For *in vitro* binding of M019 to peripheral immune cells, M019 or IgG2Fc was conjugated with Dylight488 (ThermoFisher Science) per manufacturer’s instructions and incubated with peripheral blood from healthy mice for 45 min followed by Fc blocking with anti-CD16 and anti-CD32 antibodies (Fc block; clone 2.4G2, BD biosciences). To block M019 binding, cells were incubated with anti-CD16 and anti-CD32 antibodies for 30 min prior to incubation with Dylight488-M019. Cells were incubated with antibodies to NK cells, T cells, B cells and monocytes and the percentage of immune cells binding M019 was determined for phenotype.

For *in vivo* binding studies, M019 was labeled with CF680 (Biotium, San Francisco, CA) per manufacturer’s instructions. CF680-M019 preparations with a degree of labeling between 3-5 were used. Healthy mice were injected sc with 2mg of CF680-M019, unlabeled M019 or free fluor and peripheral blood collected 24hrs later. The cells were incubated with antibodies to T cells, NK cells, B cells and monocytes and percentage of bound M019 determined for each phenotype.

### Multiplex immunoassay for cytokines and chemokines and ELISA

The levels of a panel of inflammatory mediators in brain samples were measured using a 26-plex ProcartaPlex™ Multiplex Immunoassay according to manufacturer’s instructions (Invitrogen, Carlsbad CA, USA). Cytokine standards were prepared to determine the concentration of cytokines/chemokines in the samples. The samples were run on a Luminex 100/200 instrument and analyzed with xPONENT 4.2 software. For data analysis, a five-parameter logistic curve fitting method was applied to the standards and the sample concentrations extrapolated from the standard curve.

To measure IL-17 and IFNγ in culture supernatants we used cytokine specific ELISA’s (Biolegend). Spleen cells from M019 or vehicle treated EAE induced mice or control mice were stimulated with 15µg/ml of MOG_35-55_ peptide, scrambled peptide (30µg/ml) or were left unstimulated. Cultures were incubated for 72hrs at 37°CC. The culture supernatants were collected and IL-17 and IFNγ were measured by ELISA. Cytokine standards were prepared to determine the concentration of cytokines and the results were read on a BioTek Cytation 5 Imaging microplate reader (Agilent, Santa Clara CA). For data analysis a five-parameter logistic curve fitting method was applied to the standards and the sample concentrations were extrapolated from the standard curve.

### qRT-PCR

Total RNA was extracted from the brain of individual mice using Trizol (Invitrogen). Contaminating genomic DNA was removed by treatment with DNA-free (Ambion, Austin, TX) according to manufacturers’ directions. Two µg of RNA was reverse transcribed into cDNA with the Promega reverse transcription system (Promega, Madison, WI). Real time PCR was performed with gene specific primers to IL-17A, IL-10, IFNγ, CCL7, CXCL10, and IL-18 (**Supplemental Table 2**) using a LightCycler 480 real time PCR thermal cycler (Roche Diagnostics, Indianapolis, IN). All primers and probes were chosen using the online software Universal Probe Library. All primers were purchased from Integrated DNA Technologies, Inc. (Coralville, IA). Data are normalized for GAPDH and plotted as fold induction over control mice that have not been induced with MOG/CFA.

### Histology

Spinal cord and brain were removed and fixed with neutral buffered formalin and embedded in paraffin. Eight-micron sections were cut and stained with Hematoxylin and Eosin to analyze inflammation or Luxol Fast Blue to analyze demyelination. Slides were digitized (Scanscope XT, Aperio, Vista, CA) and analyzed in a blinded manner using CaseViewer v2.4 software (HisTech Ltd). The area of the spinal cord was manually traced, and area calculated. Regions of myelination were also manually traced, and the percentage of myelination determined for each section (27).

### SDS PAGE electrophoresis

Analysis of M019 by SDS-PAGE was performed using a 3-8% NuPAGE Tris Acetate 3-8% gel (Invitrogen, Carlsbad CA) for 1h 20min at 130V using Tris Acetate SDS as running buffer as previously described (11).

### Complement ELISA

Inhibition of classical complement pathway activity was assessed using a murine specific functional complement pathway ELISA assay (Hycult Biotech, Wayne, PA) as described by the manufacturer.

### Statistical analysis

Data are expressed as mean ± S.E.M. in graphs and plots. Data were plotted in GraphPad PRISM v9.3.1 and R 4.4.2 utilizing the ggplot2-package v3.3.0 (28). Statistical significance was determined using *t*-test for comparing groups where appropriate, and permutation testing (29) (based on 100,000 permutations for each of the experiments) for the criteria of interest with respect to clinical scores, relative weight loss, disease development, and survival. P<0.05 was considered statistically significant, applied for the family-wise error rate of multiple group-wise comparisons with application of the Holm-Bonferroni sequential adjustment procedure (30).

## Results

### M019 characterization

**Figure 1:**
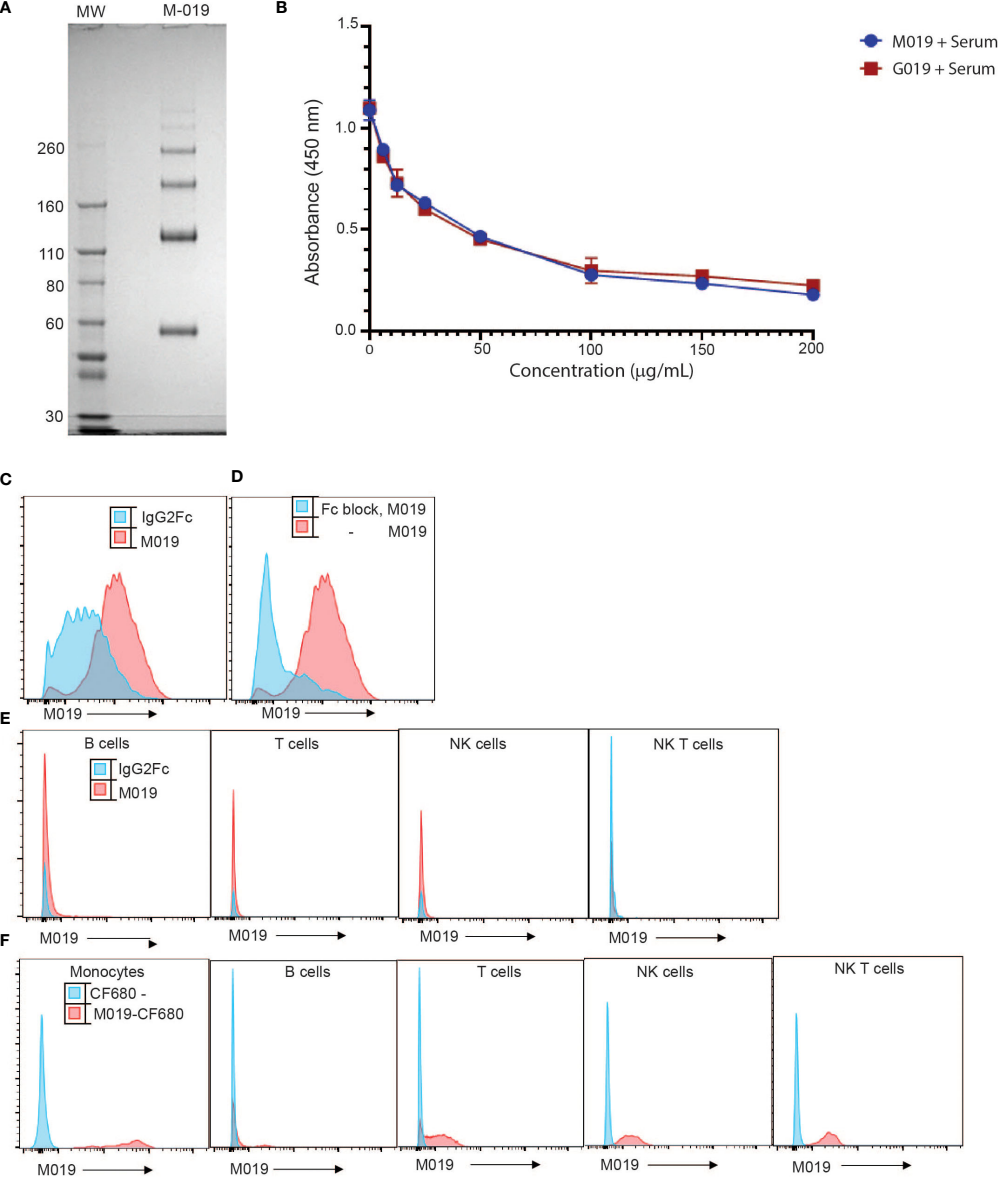
Characterization of M019 and binding to complement and immune cells. **(A)** M019 was electrophoresed using SDS-PAGE under reducing conditions. **(B)** M019 and G019 were analyzed using a functional complement ELISA. **(C)** Representative histograms demonstrating percentage of monocytes (CD11b^+^CD115^+^ cells) binding M019 (red) or the homodimer control IgG2Fc (blue). **(D)** Percentage of monocytes binding M019 following pretreatment with anti-16 and anti-CD32 antibodies (Fc block; blue). **(E)** Percentage of B cells (CD19^+^), T cells (TcRβ^+^), NK cells (NK1.1^+^ TcRβ^-^), and NK T cells (NK1.1^+^ TcRβ^+^) binding M019 *in vitro*. **F**). Representative histograms demonstrating % of peripheral blood immune cells binding M019 following *in vivo* injection. In **(C-E)** n=4-6 mice/group.

M019 consists of murine IgG2A Fc domain and incorporates a linked multimerization domain (MD) sequence from the hinge region of human IgG2 to the carboxy terminus of murine IgG2a. The IgG2 hinge region results in formation of multimers of the murine Fc domain containing both homodimers and highly ordered multimers of the Fc homodimers, the majority of which span a size range from ~ 60 to 300 kD (**Figure 1A**). Because M019 contains the CH2 and CH3 regions of IgG2a heavy chain it has potential to bind to both FcγR’s and complement components. To determine whether M019 binds to complement we measured the ability of M019 to inhibit the classical complement pathway using a functional ELISA (**Figure 1B**). Like other compounds we have developed (31), our data demonstrate that M019 inhibits complement activation as does the human Fc Multimer G019 that contains a human IgG1 Fc core, likely through C1q mediated competitive inhibition.

To determine the ability of M019 to bind to immune cells we incubated DyLight-488 labeled M019 with peripheral blood cells isolated from healthy mice (**Figure 1C**). In comparison with a homodimer DyLight488-IgG2aFc control, M019 efficiently bound to monocytes. To determine whether this binding was secondary to the engagement of low affinity FcγRs, we incubated leukocytes with anti-CD16 and anti-CD32 antibodies (Fc block) prior to M019 exposure (**Figure 1D**). These antibodies inhibited the binding of M019 to monocytes, demonstrating that M019 binds FcγRII (CD32) and/or FcγRIII (CD16). Importantly, and unlike other compounds we have previously tested, M019 did not demonstrate any binding to B cells or NK cells *in vitro* (**Figure 1E**) (12).

To determine whether M019 binding to monocytes was preserved *in vivo* (**Figure 1F**), B6 mice were injected with CF680-labeled M019 or free CF680, to measure non-specific binding of any remaining unbound CF680 in the preparation, and peripheral blood was collected 24hrs later. The binding of labeled CF680-M019 to immune cell populations was analyzed by flow cytometry with phenotypic markers for monocytes, T, B, and NK cells (**Figure 1F**). M019 effectively bound monocytes *in vivo* as measured by the increased MFI compared to free CF680. Interestingly, we also observed a low level of binding to a small population of B cells, NK cells and NK T cells. Surprisingly, there was a low level of binding observed on T cells although this wasn’t observed when the dose of labeled M019 was reduced to 1 mg. Collectively, these data indicate the M019 can influence complement activity and preferentially binds monocytes through FcγR engagement *in vitro* and *in vivo*.

### M019 inhibits EAE disease severity and demyelination in the MOG EAE model

**Figure 2:**
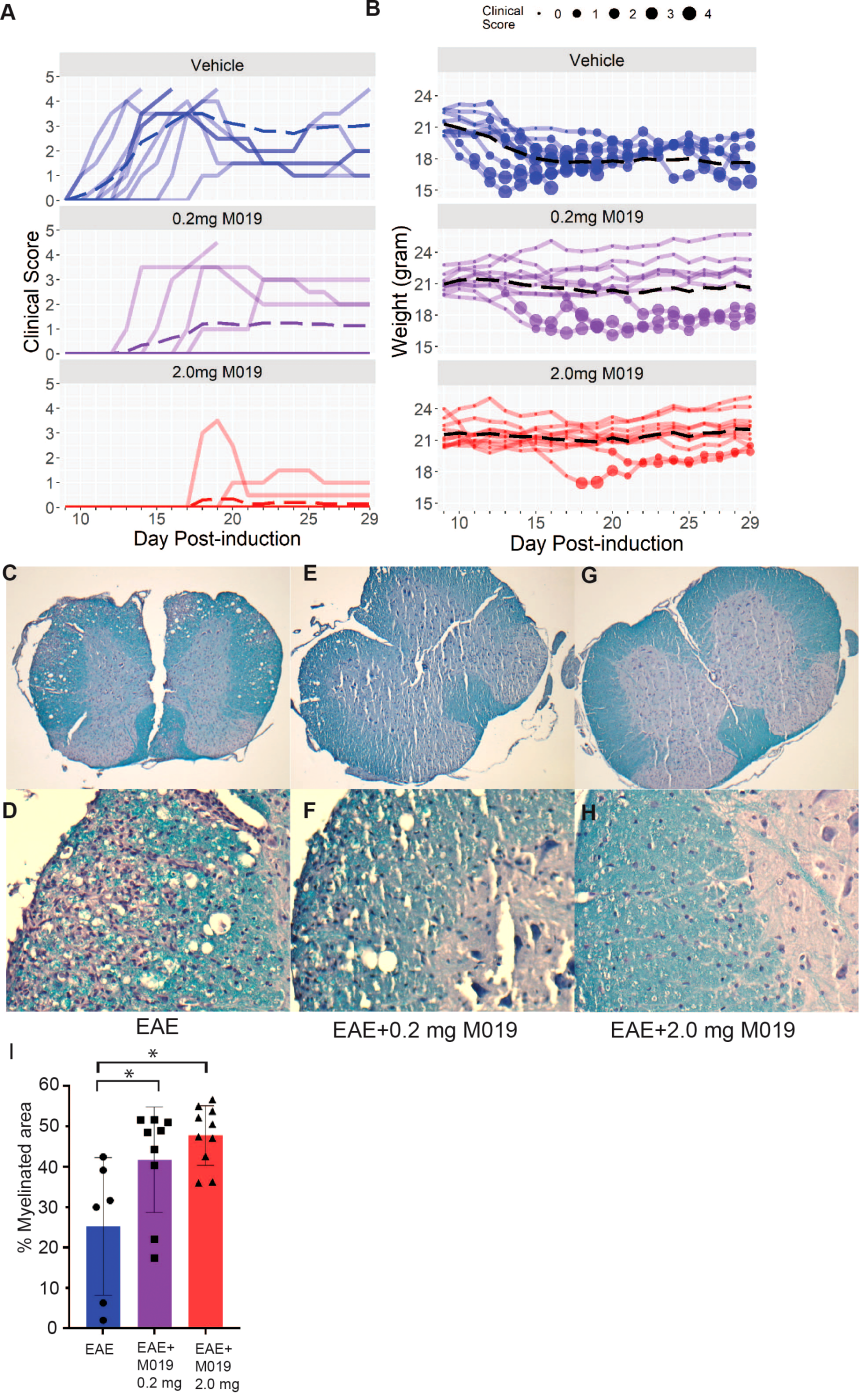
M019 reduces clinical symptoms and demyelination in the spinal cords of mice immunized with MOG. **(A)** Daily clinical scores of MOG immunized mice treated with vehicle, 0.2 mg M019 or 2.0 mg M019 beginning day 4 post-immunization. Each graph represents individual mice in the group (n=10/group). **(B)** Weights of individual mice in each group with circle size representing the clinical score. Dashed lines trace the average within each group (last observation carried forward for euthanized mice. **(C-H)** Luxol Fast blue staining of spinal cord sections from surviving mice in A at 40x magnification **(C, E, G)** or 200x magnification **(D, F, H)**. **(I)** Quantitation of myelination is expressed as mean ± S.D. *p ≤ 0.05, one way ANOVA with Tukey’s post comparison test was used to determine statistical significance.

To evaluate the potential clinical utility of Fc multimers in MS, we tested the efficacy of M019 in reducing symptoms of EAE using the C57BL/6 (B6) model of MOG-induced EAE which mimics chronic neuroinflammation. B6 mice were immunized with MOG_35-55_ peptide emulsified in CFA followed by two PTX injections, 2 and 24 hours after immunization. Beginning day 4 post-immunization, mice were treated *daily* with subcutaneous (sc) injections of M019 (0.2mg or 2mg) or vehicle through day 29 post-immunization (**Figure 2**; **Table 1**). M019 treatment reduced the proportion of mice developing disease (clinical score >1.0) from 100% (10/10), to 40% (4/10, p = 0.012) in the 0.2 mg M019 treated mice and 20% (2/10, p <.001) in the 2.0 mg M019 treated mice (**Table 1**). Importantly, M019 also significantly reduced the number of EAE mice reaching euthanasia criteria with only 50% (5/10) surviving in the vehicle treated mice, but 90% (9/10, p = 0.010) and 100% (10/10, p <.001) surviving in the 0.2 mg and 2.0 mg M019 treated mice, respectively (**Table 1**).

**Table 1:** M019 reduces disease severity in MOG induced EAE model.

	Average max. clinical score		Percent mice with clinical score >= 1		Average max. %-weight-change		Percent mice surviving	
	Observed	P-value^1^	Observed	P-value^1^	Observed	P-value^1^	Observed	P-value^1^
Vehicle (n = 10)	3.9	–	100%	–	-20.20%	–	50%	–
0.2mg M019 (n = 10)	1.45	–	40%	–	-5.69%	–	90%	–
2.0mg M019 (n = 10)	0.5	–	20%	–	-3.26%	–	100%	–
0.2mg M019 vs. Vehicle	-2.45	0.004*	-60%p^2^	0.012*	14.52%p^2,3^	0.003*	40%p^2,4^	0.010*
2.0mg M019 vs. Vehicle	-3.4	<.001*	-80%p^2^	<.001*	16.95%p^2,3^	<.001*	50%p^2,4^	<.001*
2.0mg M019 vs. 0.2mg M019	-0.95	0.285	-20%p^2^	0.276	2.43%p^2,3^	0.636	10%p^2,4^	0.595

^1^Two-sided P-value (permutation test based on 100,000 permutations).

^2^Percentage points (%p): the arithmetic difference between the percentages in the two groups.

^3^Positive number implies that first mentioned group keeps higher weight (on average).

^4^Positive number implies that first mentioned group has higher proportion of survivors (on average).

*Statistically significant on 5%-level with Holm-Bonferroni adjustment for multiplicity in pairwise comparisons (3 for each criterion).

M019 also delayed disease onset in the treated EAE mice that developed symptoms; vehicle treated mice developed clinical symptoms on day 12.9 post-immunization whereas, EAE mice treated with 0.2 mg or 2 mg of M019 developed symptoms on day 15.7 and day 19, respectively. There was a dose dependent reduction in clinical scores with average peak score of vehicle treated EAE mice = 3.9, EAE mice + 0.2 mg M019 = 1.45, and EAE mice + 2.0 mg M019 = 0.5. Furthermore, the observed differences between the 0.2 mg and 2.0 mg M019 mice compared to the vehicle treated mice were statistically significant (p = 0.004 and <.001, respectively, obtained by permutation testing with 100,000 permutations), see **Table 1** for details. Along with the reduction in clinical scores we observed a corresponding reduction in weight loss in the M019 treated mice (**Figures 2A, B**) with 14.52 (p = 0.003) and 16.95 (p <.001) percentage points (%p) less relative weight-loss in the 0.2 mg and 2.0 mg M019 treated mice, respectively. While treatment with 0.2 mg and 2.0 mg M019 on a *biweekly* basis did not prevent disease, it did reduce the number of mice reaching euthanasia criteria with only 50% (5/10) remaining in the vehicle treated mice, but 66% (6/9) and 100% (10/10) remaining in the 0.2 mg and 2.0 mg M019 treated mice, respectively (**Supplemental Figure 1**).

To determine if the reduction in clinical scores in the M019 treated mice correlated with reduced demyelination, we stained spinal cord sections from the surviving mice with Luxol Fast Blue (**Figures 2C–H**). Mice treated with either 0.2 mg or 2.0 mg of M019 had significantly less demyelination than vehicle treated mice (**Figures 2E–I**). M019 treated mice that did not develop symptoms did not show evidence of demyelination or inflammation (**Supplemental Table 1**).

### The therapeutic effects of M019 in the treatment of EAE are not strain specific

**Figure 3:**
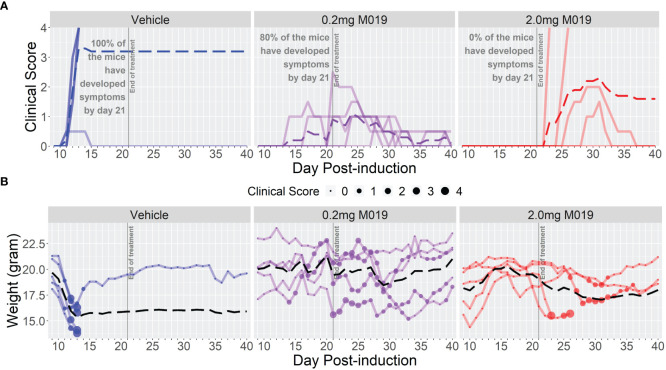
M019 reduces clinical symptoms in SJL mice immunized with PLP. **(A)** Daily clinical scores of PLP-immunized mice (n=5/group) treated with 0.2 mg or 2.0 mg of M019 twice weekly beginning day 4 and ending day 21 post-immunization (gray line). Each graph line represents an individual mouse in the group. **(B)** Weights of individual mice in each group. Dashed lines trace the average within each group (last observation carried forward for euthanized mice.

To determine if M019 could effectively reduce disease severity when EAE was induced using a different peptide and mouse strain, we tested the SJL model of relapsing remitting (RR) EAE. SJL mice were injected sc with [Ser^140^]-PLP_139-151_ emulsified in CFA on day 0 and treated with or without M019 (0.2 mg or 2.0 mg) using *biweekly* sc injections from day 4 post-immunization to day 21 post-immunization (**Figure 3**; **Table 2**). During the treatment period, M019 reduced the proportion of mice developing disease (clinical score >1.0) from 80% (4/5) in the vehicle treated mice to 0% (0/5, p=0.003) in the 2.0 mg M019 treated mice (**Table 2**). M019 treatment also significantly reduced the number of mice reaching euthanasia criteria with only 20% of vehicle treated mice surviving compared to 100% of the 0.2 mg and 2.0 mg M019 treated mice (p<0.001) surviving during the treatment period. M019 reduced disease severity in a dose dependent manner: avg max EAE score – EAE + Vehicle=3.3; EAE + 0.2 mg M019 = 1.2; EAE + 2 mg M019 = 0.0 with a statistically significant difference in the 2 mg M019 treated mice vs. vehicle (p <0.001; **Figures 3A, B**; **Table 2**).

**Table 2:** M019 reduces disease severity in PLP induced EAE model**.

	Average max. clinical score		Percent mice with clinical score >= 1		Average max. %-weight-change		Percent mice surviving	
	Observed	P-value^1^	Observed	P-value^1^	Observed	P-value^1^	Observed	P-value^1^
Vehicle (n = 5)	3.3	–	80%	–	-21.70%	–	20%	–
0.2mg M019 (n = 5)	1.2	–	80%	–	-10.51%	–	100%	–
2.0mg M019 (n = 5)	0	–	0%	–	-13.35%	–	100%	–
0.2mg M019 vs. Vehicle	-2.1	0.053	0%p^2^	0.763	11.19%p^2,3^	0.022*	80%p^2,4^	<.001*
2.0mg M019 vs. Vehicle	-3.3	<.001*	-80%p^2^	0.003*	8.35%p^2,3^	0.103	80%p^2,4^	<.001*
2.0mg M019 vs. 0.2mg M019	-1.2	0.277	-80%p^2^	0.003*	-2.84%p^2,3^	0.591	0%p^2,4^	0.743

^1^Two-sided P-value (permutation test based on 100,000 permutations).

^2^Percentage points (%p): the arithmetic difference between the percentages in the two groups.

^3^Positive number implies that first mentioned group keeps higher weight (on average).

^4^Positive number implies that first mentioned group has higher proportion of survivors (on average).

*Statistically significant on 5%-level with Holm-Bonferroni adjustment for multiplicity in pairwise comparisons (3 for each criterion).

**Includes data up to and including day 21 post-immunization, the end of M019 treatment.

Once treatment with M019 was stopped on day 21 post-immunization, the mice receiving 0.2 mg of M019 did not experience disease progression. However, mice receiving 2.0 mg of M019, which did not develop disease while on treatment, developed symptoms within 1-7 days following the last treatment. The rapid development of disease onset after treatment cessation in these mice provides presumptive evidence that M019 does not affect T cell priming. These results demonstrate that M019 reduces EAE symptoms regardless of the peptide used or the genetic background of the mice. Furthermore, they suggest that M019 does not cure the disease.

### M019 reduces disease severity and lethality when given at onset of symptoms

**Figure 4:**
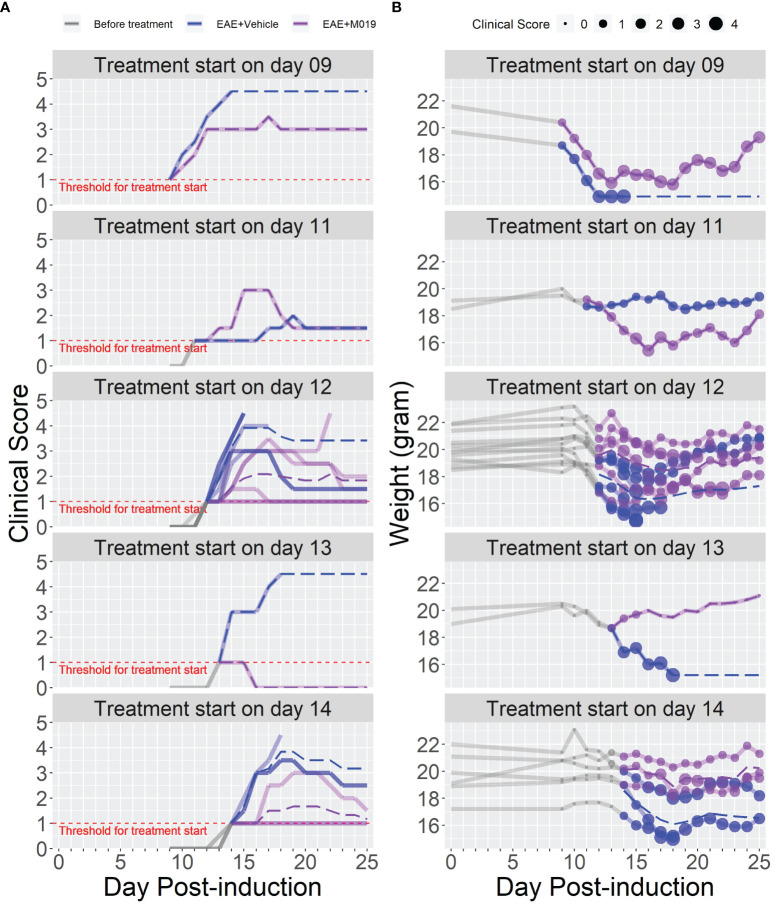
M019 reduces disease severity when given at onset of symptoms. **(A)** Daily clinical scores of MOG immunized mice randomly enrolled into M019 or vehicle treatment groups (n=12 mice/group) when they reached a clinical score of 1 on the same day. Mice were enrolled between days 9-14 post-induction and each graph panel represents mice enrolled at a specific time point. **(B)** Weights of individual mice in each group with circle size representing the clinical score. Dashed lines trace the average within each group (last observation carried forward for euthanized mice).

The previous studies demonstrate the effectiveness of M019 when given prior to symptom onset, however, MS patients would be treated with a human version of M019 after diagnosis of clinical symptoms. To measure the efficacy of M019 when given at onset of symptoms, we treated MOG immunized B6 mice with M019 (2 mg) on the day of symptom onset using a rolling enrollment strategy. Mice that reached a clinical score of 1.0 on the same day were randomly divided into M019 treated or vehicle treated groups (n=12 mice/group in total). Mice that developed symptoms were enrolled between days 9-14 post-immunization. Treatment was initiated the same day as symptom onset via sc injection and given daily until day 25 (**Figure 4**; **Table 3**). Mice treated with M019 had statistically significantly lower max clinical scores compared to mice that received vehicle only; EAE + Vehicle=3.83, EAE + 2 mg M019 = 2.25 (p=0.002, **Table 3**). In addition, there was a decrease in the number of M019 treated mice that reached a clinical score >4.0 and had to be euthanized (EAE + vehicle = 42% survived; EAE + M019 = 92% survived, p=0.002). These data demonstrate that M019 ameliorates disease severity in a therapeutic model.

**Table 3:** M019 inhibits disease severity when given at onset of symptoms.

	Average max. clinical score		Average max. %-weight-change		Percent mice surviving	
	Observed	P-value^1^	Observed	P-value^1^	Observed	P-value^1^
EAE+Vehicle (n = 12)	3.83	–	-17.27%	–	42%	–
EAE+M019 (n = 12)	2.25	–	-11.54%	–	92%	–
EAE+M019 vs. EAE+Vehicle	-1.58	0.002*	5.73%p^2,3^	0.071	50%p^2,4^	0.002*

^1^Two-sided P-value (permutation test based on 100,000 permutations).

^2^Percentage points (%p): the arithmetic difference between the percentages in the two groups.

^3^Positive number implies that first mentioned group keeps higher weight (on average).

^4^Positive number implies that first mentioned group has higher proportion of survivors (on average).

*Statistically significant on 5%-level.

### M019 reduces immune cell infiltration into spinal cord of MOG immunized mice

**Figure 5:**
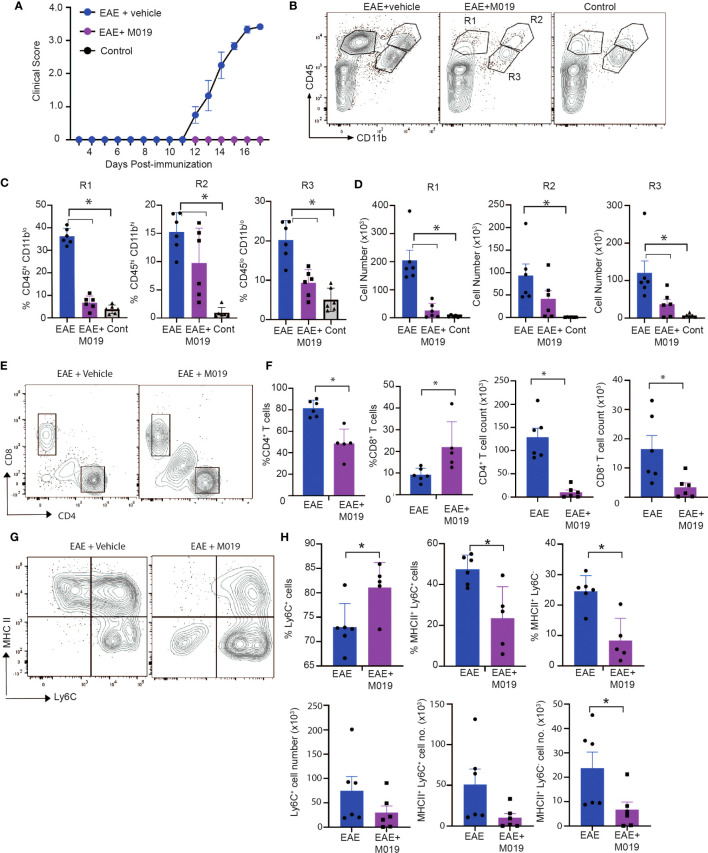
M019 reduces infiltration of immune cells into spinal cord in EAE mice. **(A)** Clinical scores of MOG immunized or control mice treated with vehicle or M019 daily beginning day 4 post-immunization. **(B)** Representative FACS plots demonstrating the % of cells expressing CD11b and CD45. **(C)** Quantitative analysis of data from B demonstrating % and **(D)** total number of cells within each gate. **(E)** Representative FACS plots of cells in the R1 gate that are TcRβ^+^ and express either CD4 or CD8. **(F)** Quantitation of FACS plots in **(E)**. **(G)** Representative FACS plot of MHC II expression on Ly6C^+^ cells in the R2 gate infiltrating spinal cords. **(H)** Quantitation of the percentages and total number of Ly6C^+^ cells and expression of MHC II. All panels represent data pooled from 2 experiments with a total n=6/group; *p ≤ 0.05, one way ANOVA with Tukey’s post comparison test was used to determine statistical significance.

Complex interactions between autoreactive lymphocytes with infiltrating and resident myeloid cells contribute to the pathology observed in spinal cords of EAE mice and ultimately disease severity. Because M019 treatment inhibited development of EAE clinical symptoms and demyelination, we speculated that there would also be reduced immune cell infiltration into the spinal cords of the treated EAE mice. We measured the level of cellular infiltration into the spinal cords of mice immunized with EAE and treated with vehicle or M019 (2mg) daily beginning day 4 post-immunization by flow cytometry. The vehicle treated immunized mice developed clinical symptoms and reached an average peak score of 3.5 by day 17 post-immunization whereas the M019 treated mice did not develop symptoms (**Figure 5A**). Overall, flow cytometric analysis of the cells in the spinal cord revealed that M019 treatment reduced the infiltration of CD45^hi^ immune cells compared to the vehicle treated mice. Using CD11b and CD45 as parental gates, we identified 3 major cell populations in the spinal cords of EAE mice; cells that were CD45^hi^ CD11b^lo^ (R1 gate) which includes lymphocytes, cells that were CD45^hi^ CD11b^hi^ (R2 gate) which contains myeloid cells such as monocytes, DCs and neutrophils and finally, cells that were CD45^int^ CD11b^+^ gate (R3 gate) which includes microglia (**Figure 5B**). As expected, there was a significant increase in both the frequency and number of cells within the R1, R2 and R3 gates in the vehicle treated EAE mice compared to control mice (**Figures 5C, D**). However, treatment with M019 significantly reduced the frequency and total cell number of lymphocytes (R1) and the frequency of myeloid cells (R2) in the spinal cord demonstrating that M019 blocked immune cell infiltration into the CNS. In addition, there was also a significant reduction in the frequency and total cell number of microglia (R3) in the M019 treated EAE mice compared to the vehicle treated EAE mice.

The pathogenicity of EAE is dependent on autoreactive CD4^+^ T cells infiltrating the CNS, whereas the role of CD8^+^ T cells is less clear, with some reports suggesting they are protective and others suggesting they contribute to disease (32, 33). Cells within the R1 gate include lymphocytes and our results demonstrate a large increase in CD4^+^ T cells in the vehicle treated EAE-induced mice that was significantly decreased in frequency and cell number by M019 treatment (**Figures 5E, F**). There was also a notable decrease in the total number of CD8^+^ T cells in the M019 treated mice compared to vehicle treated EAE-induced mice, although the percentage of CD8^+^ T cells in the M019 treated mice was significantly increased compared to the vehicle treated mice. To better to understand the nature of these cells, we assessed their expression of CD44, a marker of effector and memory cells. CD4^+^ and CD8^+^ T cells in both the vehicle and M019 treated EAE-induced mice expressed similar levels of CD44 suggesting that the small number of T cells in the CNS of M019 treated EAE-induced mice were effector T cells (data not shown).

Myeloid cells contribute significantly to EAE pathogenesis and monocyte recruitment into the CNS is critical for driving EAE pathogenesis. In the EAE model the number of pro-inflammatory monocytes increases in the circulation shortly after immunization and their migration into the spinal cord correlates with onset of clinical symptoms (10, 21). Cells within the R2 gate include monocytes, as defined by Ly6C expression, that have infiltrated the CNS. There was a non-significant decrease in the number of Ly6C^+^ cells in the M019 treated EAE-induced mice compared to the vehicle treated EAE-induced mice (**Figure 5G, H**). However, the frequency of Ly6C^+^ cells was significantly increased in the M019 treated EAE mice suggesting that it is mainly the Ly6C^+^ cells that can infiltrate the CNS during M019 treatment. To determine if M019 treatment affected molecules involved in antigen presentation in the CNS, we measured the expression of MHC II on the cells within the R2 gate (**Figures 5G, H**). M019 treatment significantly inhibited the expression of MHCII by both Ly6C^+^ and Ly6C^-^ cells compared to the vehicle treated EAE-induced mice, suggesting these cells would not be effective at reactivating infiltrating autoreactive T cells.

### M019 treatment inhibits co-stimulatory molecule expression by microglial cells following EAE induction

**Figure 6:**
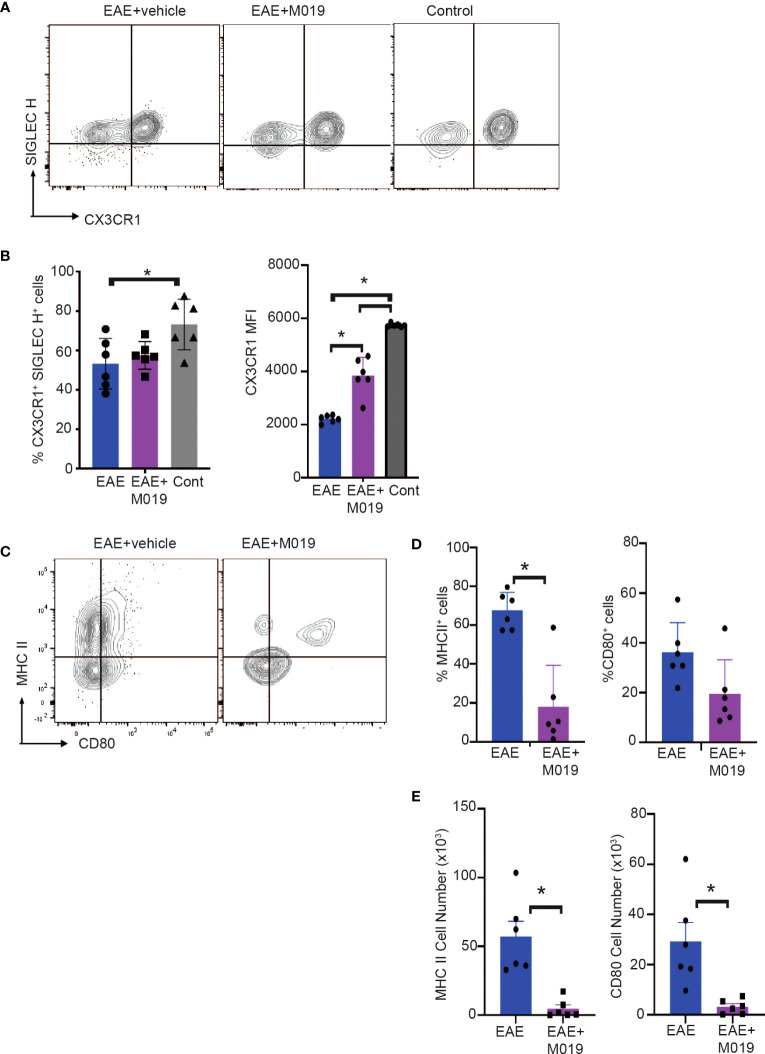
M019 reduces microglial cell activation in spinal cords of EAE mice. **(A)** Representative FACS plots of cells from R3 gate expressing SIGLEC H and CX3CR1. **(B)** Mean Fluorescence intensity of CX3CR1 expression on the CX3CR1^+^ population. **(C)** Representative FACS plots demonstrating expression of MHCII and CD80 on cells in the R3 gate. **(D)** Frequency and **(E)** total number of cells expressing MHCII and CD80. All panels represent data pooled from 2 experiments with a total n=6/group; *p<0.05, one way ANOVA with Tukey’s post comparison test was used to determine statistical significance.

Cells within the CD45^int^ CD11b^+^ gate (R3) include microglia as well as CNS-associated macrophages and infiltrating monocyte-derived macrophages which can be difficult to distinguish (34–37). We further analyzed cells within this gate for expression of the microglial markers, SIGLEC H and CX3CR1 (**Figure 6A**). There was a significant decrease in CX3CR1^+^ SIGLEC H^+^ cells in the vehicle treated EAE mice compared to the control mice (**Figure 6B**). However, this decrease was not significant in the M019 treated mice. CX3CR1 expression on microglia decreases during peak EAE (38) and we observed a significant decrease in CX3CR1 expression in the vehicle treated EAE mice (as measured by MFI) compared to the M019 treated and control groups (**Figure 6B**). However, there was also a significant decrease in CX3CR1 expression in the M019 treated mice compared to the control group suggesting M019 did not completely prevent its downregulation. Analysis of co-stimulatory markers on cells within the R3 gate demonstrated that there was a significant increase in the frequency of cells expressing MHCII in the vehicle treated EAE-induced mice (67.5 ± 9.5%) compared to M019 treated EAE mice (18.0 ± 21.2%; **Figures 6C–E**). Although the frequency of cells expressing CD80 in the R3 gate was not different between vehicle and M019 treated mice there was a significant difference in the number of CD80^+^ cells between the two groups of mice (**Figures 6D, E**). These results suggest that M019 limits the ability of microglia to present antigen to autoreactive T cells following MOG immunization.

### M019 does not alter the frequency or number of myeloid cell populations in the periphery

The reduced numbers of myeloid cells infiltrating the CNS of M019 treated mice from the periphery may be due to cell death induced by M019 binding. We analyzed the monocyte and neutrophil populations in the circulation and spleen at 7- and 13-days post-induction (**Table 4**). The percentage of neutrophils in the blood of M019 and vehicle treated mice was significantly increased in comparison to control mice at both timepoints. However, at day 7 post-induction the vehicle treated mice had double the percentage of neutrophils as the M019 treated mice. By day 13 post-induction, both the M019 and vehicle treated mice had a similar frequency of neutrophils. The percentage of inflammatory monocytes (Ly6C^+^ CD11b^+^ Ly6G^-^) in the blood was not significantly different in the M019, vehicle treated, or control groups at day 7 post-induction. However, there was a significant increase in the percentage of Ly6C^+^ monocytes in the M019 treated mice compared to vehicle treated and control mice by Day 13. Within the spleen there was no difference in the percentage or number of neutrophils or Ly6C^+^ monocytes between the M019 and vehicle treated mice although both were significantly increased compared to the control mice at both time points. There were also significant differences in the frequencies of CD4^+^ T cells, CD8^+^ T cells and B cells in the M019 and vehicle treated mice compared to the control mice. However, M019 treatment did not alter those frequencies and there was no difference in cell numbers between any of the groups.

**Table 4:** M019 treatment does not reduce circulating or splenic myeloid cells following EAE induction.

		Day 7	Day 13
Blood			
**% PMNs** **(Ly6G^+^ CD11b^+^)**	EAE	17.8 ± 9.4	31.0 ± 14.4*
	EAE+M019	8.9 ± 5.4	32.3 ± 9.4*
	Control	1.6 ± 0.8	6.7 ± 1.9
**% Inflammatory monocytes** **(Ly6C^+^ CD11b^+^ Ly6G^-^)**	EAE	3.9 ± 0.7	12.6 ± 7.4**
	EAE+M019	7.2 ± 3.0	20.5 ± 2.4**
	Control	5.1 ± 1.8	4.5 ± 0.8
Spleen			
**% PMNs** **(Ly6G^+^ CD11b^+^)**	EAE	13.2 ± 1.9	8.6 ± 2.5*
	EAE+M019	8.7 ± 2.7	8.3 ± 1.5*
	Control	0.93 ± 0.17	0.22 ± 0.02
**PMN cell number**	EAE	1.0x10^7^ ± 0.4	7.0125x10^6^ ± 2.18*
	EAE+M019	0.9 x10^7^ ± 0.3	6.388 x10^6^ ± 1.83*
	Control	0.04 x10^7^ ± 0.001	0.120 x10^6^ ± 0.0027
**% Inflammatory monocytes** **(Ly6C^+^ CD11b^+^ Ly6G^-^)**	EAE	2.4 ± 0.59	4.4 ± 0.5**
	EAE+M019	3.0 ± 0.08	5.2 ± 0.27**
	Control	1.4 ± 0.26	1.04 ± 0.18
**Inflammatory monocyte cell number**	EAE	2.002x10^6^ ± 0.75	3.595x10^6^ ± 0.62*
	EAE+M019	3.016x10^6^ ± 0.26	4.165x10^6^ ± 1.56*
	Control	0.603x10^6^ ± 0.008	0.437x10^6^ ± 0.24
**% CD4^+^ T cell**	EAE		12.6 ± 1.3*
	EAE+M019		11.7 ± 2.3*
	Control		15.6 ± 1.9
**CD4^+^ T cell number**	EAE		1.02x10^7^ ± 0.19
	EAE+M019		0.94 x10^7^ ± 0.39
	Control		0.86x10^7^ ± 0.25
**% CD8^+^ T cell**	EAE		5.8 ± 1.0*
	EAE+M019		5.3 ± 1.8*
	Control		9.2 ± 0.6
**CD8^+^ T cell number**	EAE		0.47x10^7^ ± 0.13
	EAE+M019		0.43x10^7^ ± 0.23
	Control		0.50x10^7^ ± 0.11
**% B cells**	EAE		41.4 ± 5.8*
	EAE+M019		37.6 ± 4.2*
	Control		61.5 ± 1.6
**B cell number**	EAE		3.36 x10^7^ ± 0.66
	EAE+M019		2.99 x10^7^ ± 1.1
	Control		3.30 x10^7^ ± 0.74

Frequency of cell populations are expressed as average percentage of the cell population with respect to total population. * P<0.05 compared to control, ** P<0.05 between M019 and vehicle treated groups; two-way ANOVA followed by Tukey’s post-comparison test.

We also measured splenic hypertrophy in the M019 and vehicle treated EAE induced mice. Spleen weights from vehicle treated EAE induced mice were significantly increased in comparison to control mice (EAE spleen weight = 0.169 g ± 0.015; control spleen weight = 0.084 g ± 0.008; p=0.0008; One-way ANOVA). However, there was no significant difference in the spleen weights between the vehicle treated and M019 treated EAE induced mice (EAE spleen weight = 0.169 g ± 0.015; M019 treated EAE spleen weight = 0.167 g ± 0.011; p=0.9932). Taken together, these data suggest that the decrease in cell infiltration into the CNS was not due to a depletion of these cell populations in the periphery. The increased frequency of monocytes in the peripheral blood of the M019 treated mice provides additional support for the idea that these cells might be sequestered in the periphery.

### M019 does not alter Th17 or Treg cell numbers

**Figure 7:**
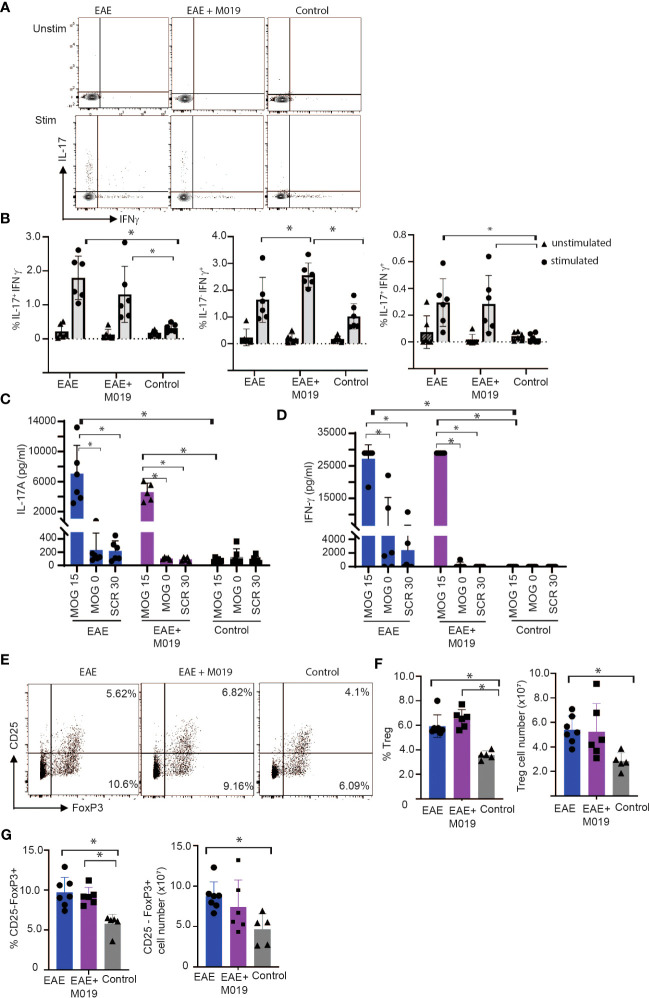
M019 does not affect Th17 development in EAE mice. **(A)** Representative FACS plots of intracellular cytokine staining of spleen cells from EAE mice treated with vehicle or M019 daily beginning day 5 post-immunization. **(B)** Quantitation of Th17, Th1 or double positive cells. Panels represent data pooled from 2 experiments with a total n=6/group; **(C)** Quantitation of IL-17 and IFNγ **(D)** in culture supernates from MOG stimulated spleen cells. **(E)** Representative FACS plots of Treg cells (TcRβ^+^ CD4^+^ FoxP3^+^ CD25^+^) in spleens of EAE mice treated with vehicle or M019 daily. **(F, G)** Frequency and number of Treg cells in spleen. Panels represent data from n = 6-7 mice/group; *p ≤ 0.05, two-way ANOVA with Tukey’s post-comparison test was used to determine statistical significance.

It is conceivable that M019 might function by working on Fc bearing APCs in the periphery to limit differentiation of Th17 cells. To determine the extent to which M019 limits Th17 cell generation following EAE induction, we stimulated spleen cells from vehicle and M019 treated EAE induced mice with anti-CD3 and anti-CD28 antibodies and measured IL-17 and IFNγ cytokine expression by ICC (**Figures 7A, B**). The results demonstrate that compared to the stimulated cells from control mice, there was a significant increase in IL-17 single positive and IL-17, IFNγ double positive CD4^+^ T cells in both the vehicle and M019 treated EAE induced mice demonstrating the M019 treatment did not inhibit Th17 cell generation (**Figure 7B**). Interestingly, the frequency of IFNγ expressing CD4^+^ T cells was increased in the M019 treated EAE induced mice compared to the vehicle treated and control mice.

To demonstrate that the response was MOG specific, we stimulated spleen cells from both vehicle and M019 treated EAE induced mice with MOG_35-55_ peptide, scrambled peptide or media alone and measured IFNγ and IL-17 in the culture supernatants after 72hrs (**Figures 7C, D**) The results demonstrate that there was an increase in both IL-17 and IFNγ in MOG_35-55_ stimulated cells from both M019 and vehicle treated EAE induced mice in comparison to the control mice. The response was specific for MOG_35-55_ peptide because there was no response to the scrambled peptide control. These data suggest that M019 treatment did not affect the generation of a Th17 response in the periphery.

To determine if M019 induced an increase in Treg cells we measured the frequency and number of Tregs (defined by CD4^+^ CD25^+^ FoxP3^+^) in the spleens of M019 and vehicle treated EAE induced mice (**Figures 7E, F**). The results demonstrate that although both groups have a significant increase in the percentage of splenic Tregs compared to the control mice, there was no significant difference between the M019 treated and vehicle treated EAE mice. Additionally, there was no significant difference in the number of Tregs in the spleen of M019 or vehicle treated EAE mice. We also measured the frequency and cell number of CD4^+^ CD25^-^ FoxP3^+^ cells which are reported to be less suppressive than conventional Tregs (**Figure 7G**) (39, 40). Both M019 and vehicle treated EAE induced mice exhibited a significant increase in the frequency of these cells compared to control mice. However, there was also no difference in the frequency or cell number of these cells between the M019 and vehicle treated mice. These results suggest that the lack of clinical symptoms and immune cell infiltration is not due to an increase in Treg cells in the M019 treated mice.

### M019 alters chemokine expression patterns in MOG immunized mice

**Figure 8:**
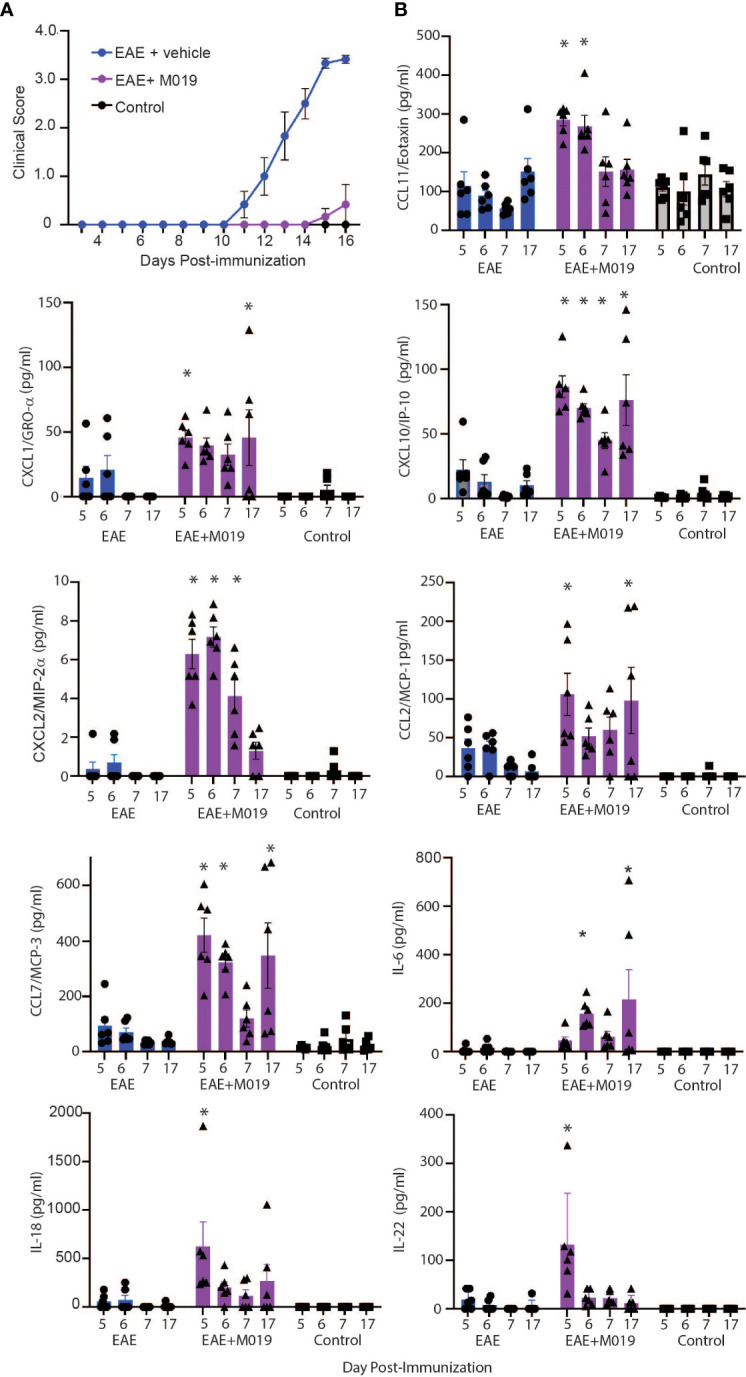
M019 treatment results in increased chemokine levels in the sera of EAE mice. **(A)** Daily clinical scores of MOG immunized mice treated with vehicle or 2.0 mg M019 beginning day 4 post-immunization. Data represents that avg ± S.E.M. **(B)** Chemokine levels in the sera of mice measured with a multiplex immunoassay. All panels represent data pooled from 2 experiments with a total n=6/group; *p ≤ 0.05, two-way ANOVA followed by Tukey’s post-comparison test.

Based on our observations that most animals treated with M019 did not develop inflammatory infiltrates in the CNS yet were not deficient in Th17 cell generation in the spleen, we postulated that M019 might influence trafficking of immune cells into the CNS through alterations in cytokine and/or chemokine levels. We used a 26-plex immunoassay to measure the level of cytokines and chemokines in the serum of MOG immunized mice treated with vehicle or M019 daily beginning on day 4 post-immunization; the serum was collected on days 5, 6, 7 and 17 post-EAE induction (**Figure 8**). In this experiment, the disease incident rate at day 17 post-immunization in the EAE + vehicle group was 100% and 33% in the EAE + M019 treatment group. The average clinical score of mice with symptoms at day 17 in the EAE + Vehicle group = 3.5 ± 0 and in the EAE + M019 treated group was 1.25 ± 0.75 (**Figure 8A**). Neither M019 treated nor vehicle treated EAE induced mice exhibited increased levels of anti-inflammatory or Th2 associated cytokines such as IL-10, IL-4, IL-5, IL-9, or IL-13 (data not shown). There was an increase in IL-18 and IL-22 in the M019 treated EAE induced mice on day 5 and an increase in IL-6 on days 6 and17 post-induction compared to vehicle treated and control mice. We did not detect GM-CSF, IL-2, IL-23, IL-12p70, IL-17A, IL-27, or IL-1β in any of the serum samples (data not shown).

In contrast to the cytokine results, several chemokines were expressed at significantly different levels between the vehicle and M019 treated EAE mice. The chemokines CXCL10/IP-10, CCL7/MCP-3, and CXCL2/MIP-2α were all increased in the serum of M019 treated mice compared to the vehicle treated EAE induced mice, in at least 3 of the timepoints tested (**Figure 8B**). M019 treatment increased the levels of CXCL1/Gro-α and CCL2/MCP-1 on days 5 and 17 post-induction and CCL11/Eotaxin on days 5 and 6 post-induction compared to the vehicle treated EAE induced mice and control mice. We did not detect MIP-1α (CCL3), RANTES (CCL5), or MIP-1β (CCL4) in any of the serum samples. To determine if there was differential regulation of cytokines or chemokines in the CNS, we analyzed mRNA expression in brains of mice at day 7 and 17 post-induction using qRT-PCR (**Supplemental Figure 2**). The results demonstrate that by day 17 post-immunization, the level of IFNγ mRNA was significantly increased in the vehicle treated EAE mice in comparison to the M019 treated EAE mice. However, there was no significant difference in the levels of CCL7/MCP-3 and CXCL10/IP-10 between the vehicle and M019 treated groups; although they exhibited a trend towards reduced levels in the M019 treated mice.

**Figure 9:**
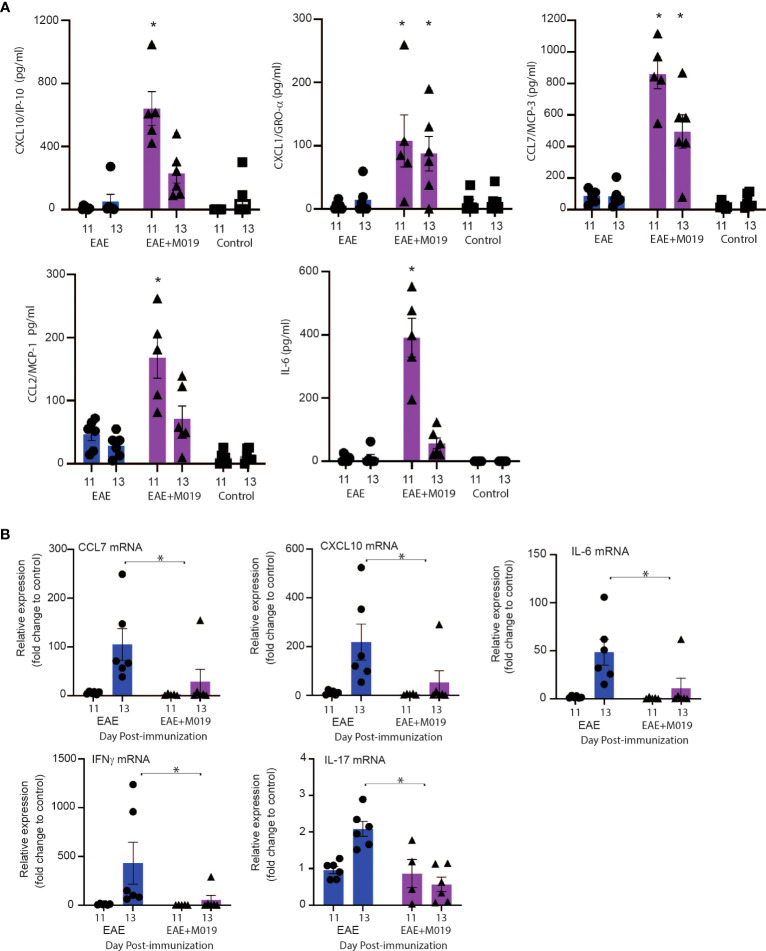
Cytokine and chemokine mRNA expression in spinal cord of M019 and vehicle treated EAE induced mice. Cytokine and chemokine levels in the sera of EAE mice treated with and without M019 at days 11 and 13 post-immunization **(A)**. Relative expression of cytokine and chemokine mRNA in the spinal cord from M019 or vehicle treated mice normalized to the housekeeping gene HPRT and expressed as fold induction over control mice **(B)**. All panels represent data pooled from 2 experiments with a total n=6/group; *p ≤ 0.05, Data analyzed using Mann-Whitney test.

To confirm that M019 altered chemokine levels in the sera and spinal cord at the time of symptom onset, we measured their levels in the sera by multiplex immunoassay and in the spinal cord by qRT-PCR at days 11 and 13 post-immunization (**Figure 9**). There was a significant increase in CXCL10, CCL7, CXCL1 and CCL2 in the sera of M019 treated EAE mice at day 11 post-immunization compared to the vehicle treated EAE and control mice (**Figure 9A**). We did not detect IFNγ or IL-17 in the sera, however there was a significant increase in IL-6 in the sera of M019 treated mice compared to the vehicle treated mice. Conversely, there was a significant reduction in CCL7 and CCXCL10 mRNA expression in the spinal cord, as well as the pro-inflammatory cytokines IL-6, IFNγ, and IL-17 in the M019 treated mice compared to the vehicle treated EAE mice (**Figure 9B**). These results suggest that there is a decrease in inflammatory cytokine and chemokine production in the CNS despite the increased chemokine levels in the serum, suggesting that altered migration patterns may contribute to the therapeutic activities of M019.

## Discussion

We demonstrate that the Fc multimer, M019, inhibits EAE associated clinical symptoms and demyelination in both the relapsing remitting and chronic inflammation EAE mouse models and these effects are observed even after the onset of clinical symptoms. Our data suggests the abrogation of disease activity is not secondary to a decrease in Th17 cells particularly since clinical symptoms develop shortly after cessation of treatment. Rather, M019 prevents migration of myeloid and lymphoid cells into the CNS with associated reduction in the expression of co-stimulatory molecules on microglia. While the mechanism(s) responsible for these trafficking changes remain uncertain, the limited infiltration into the CNS of M019 treated animals is associated with distinct patterns of chemokine upregulation in the sera. These results suggest that M019 inhibits the trafficking of pathogenic cells into the CNS of mice with EAE and might offer a new approach for the treatment of MS and potentially other neuroinflammatory diseases.

Patients with MS manifest a spectrum of clinical symptoms (3, 4) and our studies demonstrated that M019 effectively reduced disease severity in terms of clinical scores and demyelination in the spinal cord in the B6 model of chronic inflammation and the SJL model of relapsing remitting EAE when given at day 4 post-immunization. In both models, the incidence rate was reduced, and the day of onset delayed in a dose dependent manner, suggesting that the therapy may be effective in patients with different forms of the disease. The SJL model required a lower amount of M019 to reduce disease severity compared to the B6 model (twice weekly vs daily injections). While there are several potential explanations for the improved potency of M019 in SJL mice, e.g. the role of specific chemokines differs between the SJL and B6 EAE models [reviewed in (41)], we did not address these in the present study. These results suggest that the Fc multimers may benefit patients with heterogeneous clinical symptoms, even after the onset of disease.

M019 significantly inhibited the infiltration of CD4^+^ and CD8^+^ T cells into the CNS without altering their numbers of frequencies in the periphery. Several lines of evidence suggest that this effect did not result from an inability to generate a Th17 cell response. For example, our studies in SJL mice revealed that they developed disease within days of stopping M019 treatment. This rapid course of disease onset suggests that antigen specific T cells were present in the periphery and poised to enter the CNS upon cessation of M019 dosing. Furthermore, intracellular cytokine staining demonstrated no differences in the frequency of Th17 cells (IL-17 single positive or IL-17^+^ IFNγ^+^ double positive) in the spleens of M019 treated mice compared to the vehicle treated mice following ex vivo polyclonal stimulation. Studies have suggested that Th17 cells expressing both IL-17 and GM-CSF are pathogenic in EAE (42) (43), however we were unable to detect GM-CSF expression in CD4^+^ T cells in either of these groups. There was a significant increase in the frequency of Th1 cells in the M019 treated mice compared to the vehicle treated mice. Studies have suggested that Th1 cells are critical for allowing entry of Th17 cells into the CNS during EAE and our results demonstrate that M019 treatment did not inhibit the generation of these cells when given prior to the onset of symptoms (44). There was no evidence of an increase in Th2 cells (e.g. no increase in IL-4). MOG_35-55_ stimulation of the spleen cells resulted in increased IL-17 and IFNγ in the culture supernatants in comparison to stimulation with scrambled peptide suggesting that the response was antigen specific. Additionally, we did not detect an increase in the frequency of conventional Treg cells or CD25^-^ FoxP3^+^ Treg cells in the spleens of M019 treated mice compared to the vehicle treated mice, although both were increased compared to control mice. It has been reported that the level of suppressive activity of Tregs correlates with the level of CD25 expression; cells expressing higher levels of CD25 exhibit the most suppressive activity (45). Although we did not measure the suppressive function of either of these cell populations, the lack of change in cell number or frequency with M019 treatment, particularly with the CD25^+^ Foxp3^+^ population, suggests that M019 is not inhibiting disease through increased induction of Treg cells. These results suggest that M019 does not act as a general immunosuppressant or prevent Th1 or Th17 cell development, but effectively prevents their migration into the CNS.

We postulate that because T cells lack FcγRs, M019 inhibition of T cell migration into the CNS is likely mediated through an FcγR rich population of monocytic lineage. This hypothesis is supported by our binding studies, demonstrating that monocytes are a major cellular target for M019 both *in vivo* and *in vitro*. Furthermore, M019 treatment reduced myeloid cell infiltration into the CNS and those cells that were present expressed lower levels of MHC class II, likely limiting their ability to present antigen to autoreactive T cells. The failure of myeloid cells to migrate into the CNS is not due M019-triggered cell depletion. We did not observe any significant decrease in the inflammatory (Ly6C^+^) monocyte population or neutrophils in either the blood or spleen at day 7 or day 13 post-induction. In fact, we observed a significant increase in the frequency of Ly6C^+^ monocytes in the blood at day 13 suggesting that they are sequestered in the periphery.

Monocytic infiltration of the CNS is critical for driving disease pathogenesis in the B6 model of EAE. The number of pro-inflammatory monocytes increases in the circulation shortly after immunization and their migration into the spinal cord correlates with onset of clinical symptoms (46). Depletion of monocytes with chlodronate liposomes suppresses the development of EAE and CCR2 KO mice do not develop EAE demonstrating the critical role that monocytes play in the disease (23, 24, 47). Once in the CNS, the Ly6C^+^ CCR2^+^ monocytes are necessary for reactivation of autoreactive Th17 cells and disease pathogenesis (26). Therefore, the reduced numbers of Ly6C^+^ monocytes in combination with the reduced MHCII expression observed in the M019 treated mice likely contributes to the decreased severity of EAE.

The role of microglia in antigen presentation to autoreactive T cells is unclear. Studies have demonstrated they can take up myelin and express MHCII and co-stimulatory molecules (48, 49). However, other studies have demonstrated that MHCII expression by microglia is not necessary for disease pathogenesis or T cell priming (50, 51). We observed a significant decrease in MHCII expression by cells in the microglia gate (R3) from mice that had been treated with M019 compared to the vehicle treated mice. Additionally, M019 treatment preserved expression of CX3CR1 on the SIGLEC H^+^ microglia population compared to the vehicle treated EAE mice which exhibited reduced expression of CX3CR1. CX3CR1 is a critical neuromodulator that contributes to neurogenesis and neuroprotection. CX3CR1 expression has been reported to be decreased on microglia during peak EAE (38) and induction of EAE in *cx3cr1^-/-^* mice results in a significant increase in disease severity, demyelination and neurological deficiencies (52). It is unclear whether M019 can enter the CNS during different stages of the disease process. In healthy mice it is unlikely that the larger aggregates in M019 can cross the blood brain barrier (BBB) which also restricts IgG entry. However, once the disease is initiated and there is loss of BBB integrity it is possible that M019 may enter the CNS and bind to FcγRs which are known to be expressed by microglia (53). Collectively, these data suggest that M019 is either directly or indirectly affecting microglia phenotype and conceivably their function.

The lack of immune cell infiltration in M019 treated animals suggested that it might function by altering expression of specific cytokine/chemokine pathways that are necessary for cell recruitment into the CNS. Somewhat to our surprise, cytokine and chemokine multiplex data revealed that the M019 treated mice had *higher* levels of the chemokines CXCL10, CCL11, CCL2, CXCL2, and CCL7 in the serum compared to the vehicle treated and control mice. The identity of the cells producing the chemokines in the serum is unclear. Many of these chemokines are produced by T cells as well as myeloid cells and we are currently working to identify the cell source. There was also an increase in the levels of IL-6, IL-18, and IL-22 at specific timepoints in the sera of M019 treated mice compared to vehicle treated EAE mice. However, we did not observe the upregulation of any anti-inflammatory cytokines such as IL-10 suggesting that the mechanism of action does not rely on increased anti-inflammatory molecules.

These results differ from studies using a different Fc multimer, GL2045 which induced expression of IL-10 and reduced IL-17 and IL-6 expression (12). These differences may be due to different model systems and/or timing of sample collection in relationship to drug dosing. In contrast, we did not observe a significant increase in CXCL10 or CCL7 mRNA in the Spinal cords of the M019 treated mice in comparison to the vehicle treated mice instead there was a trend towards lower levels of these chemokines in the CNS. Additionally, there was a significant decrease in the level of IFNγ in the CNS of M019 treated mice suggesting that M019 reduced the inflammatory environment in the CNS when given prior to the onset of symptoms.

Several of the chemokines expressed at higher levels in M019 treated mice are important for monocyte and T cell recruitment. For instance, CCL2 and CCL7 both bind to CCR2 expressed on Ly6C^+^ monocytes which are critical for driving EAE pathogenesis (25). Similarly, CCL2 and CXCL10 are among the chemokines upregulated in the CNS during the acute stages of EAE and levels of CCL2 and CCL5 in the CNS have been shown to correlate with disease severity. However, there is also some evidence that these chemokines may play a protective role. CXCL10 binds to CXCR3 and is critical for recruiting T and NK cells. C57Bl/6 mice deficient in CXCR3 exhibit increased EAE disease severity which may be due to reduced Treg cell migration and unrestrained spatial organization (54). Future studies will identify the cells producing these chemokines.

While naturally occurring and rICs are recognized to inhibit ADCC, ADCP and CDC, to the best of our knowledge, this is the first report showing that a recombinant Fc multimer designed to mimic the anti-inflammatory properties of ICs inhibit migration of both myeloid and lymphoid cells into the CNS. Our working model is that M019 engages FcγR rich monocytic cells altering their activation status, migratory capacity and patterns of chemokine secretion. These changes inhibit the migration of pathogenic T cells into the CNS with resultant abrogation of EAE. An important caveat to this is that these studies were performed using daily dosing that began prior to onset of clinical symptoms. To develop Fc multimers as a treatment for MS, it is imperative to test our hypothesis using a therapeutic dosing strategy. Future studies to validate/refute this model with dosing starting after the onset of clinical symptoms is need for determining if myeloid cells are necessary/sufficient for M019 anti-inflammatory activity, defining whether M019 acts on myeloid cell populations centrally and/or peripherally, and determining if there is a direct effect of M019 on autoreactive T cell priming.

## Data availability statement

The raw data supporting the conclusions of this article will be made available by the authors, without undue reservation.

## Ethics statement

The animal studies were approved by Institutional Animal Care and Use Committee University of Tennessee Health Care Center. The studies were conducted in accordance with the local legislation and institutional requirements. Written informed consent was not obtained from the owners for the participation of their animals in this study because mice were purchased from approved vendor.

## Author contributions

JW and KB performed *in vivo* experiments and analyzed data, CD and SR helped with experiments, EM performed the complement assay and gel electrophoresis, SG production and purification of M019, KT analyzed and provided blinded scoring of the histology, FT provided statistical analysis, DB and HO provided material support, EF and SS contributed to conceptualization, data interpretation and supervision of the study. All authors contributed to the article and approved the submitted version.

## References

[1] Lassmann H Bruck W Lucchinetti CF. The immunopathology of multiple sclerosis: an overview. Brain Pathol (2007) 17:210–8. doi: 10.1111/j.1750-3639.2007.00064.x PMC8095582 17388952

[2] Milo R Miller A. Revised diagnostic criteria of multiple sclerosis. Autoimmun Rev (2014) 13:518–24. doi: 10.1016/j.autrev.2014.01.012 24424194

[3] Lublin FD Reingold SC. Defining the clinical course of multiple sclerosis: results of an international survey. National Multiple Sclerosis Society (USA) Advisory Committee on Clinical Trials of New Agents in Multiple Sclerosis. Neurology (1996) 46:907–11. doi: 10.1212/WNL.46.4.907 8780061

[4] Weinshenker BG Bass B Rice GP Noseworthy J Carriere W Baskerville J. The natural history of multiple sclerosis: a geographically based study. I. Clinical course and disability. Brain (1989) 112(Pt 1):133–46. doi: 10.1093/brain/112.1.133 2917275

[5] Gasperini C Cefaro LA Borriello G Tosto G Prosperini L Pozzilli C. Emerging oral drugs for multiple sclerosis. Expert Opin Emerg Drugs (2008) 13:465–77. doi: 10.1517/14728214.13.3.465 18764723

[6] Rio J Nos C Tintore M Tellez N Galan I Pelayo R. Defining the response to interferon-beta in relapsing-remitting multiple sclerosis patients. Ann Neurol (2006) 59:344–52. doi: 10.1002/ana.20740 16437558

[7] Wieland A Shashidharamurthy R Kamphorst AO Han JH Aubert RD Choudhury BP. Antibody effector functions mediated by Fcgamma-receptors are compromised during persistent viral infection. Immunity (2015) 42:367–78. doi: 10.1016/j.immuni.2015.01.009 PMC4339104 25680276

[8] Yamada DH Elsaesser H Lux A Timmerman JM Morrison SL de la Torre JC. Suppression of Fcgamma-receptor-mediated antibody effector function during persistent viral infection. Immunity (2015) 42:379–90. doi: 10.1016/j.immuni.2015.01.005 PMC4334737 25680277

[9] Tankersley DL Preston MS Finlayson JS. Immunoglobulin G dimer: an idiotype-anti-idiotype complex. Mol Immunol (1998) 25(1):41–8. 10.1016/0161-5890(88)90088-0 3343971

[10] Teeling JL Jansen-Hendriks T Kuijpers TW de Haas M van de Winkel JG Hack CE. Therapeutic efficacy of intravenous immunoglobulin preparations depends on the immunoglobulin G dimers: studies in experimental immune thrombocytopenia. Blood (2001) 98(4):1095–9. 10.1182/blood.v98.4.1095 11493456

[11] Jain A Olsen HS Vyzasatya R Burch E Sakoda Y Merigeon EY. Fully recombinant IgG2a Fc multimers (stradomers) effectively treat collagen-induced arthritis and prevent idiopathic thrombocytopenic purpura in mice. Arthritis Res Ther (2012) 14:R192. doi: 10.1186/ar402 22906120 PMC3580588

[12] Zhang X Owens J Olsen HS So E Burch E McCroskey MC. A recombinant human IgG1 Fc multimer designed to mimic the active fraction of IVIG in autoimmunity. JCI Insight (2019) 4. doi: 10.1172/jci.insight.12190 PMC6413835 30674715

[13] Thiruppathi M Sheng JR Li L Prabhakar BS Meriggioli MN. Recombinant IgG2a Fc (M045) multimers effectively suppress experimental autoimmune myasthenia gravis. J Autoimmun (2014) 52:64–73. doi: 10.1016/j.jaut.2013.12.014 24388113 PMC4518541

[14] Abreu SL. Suppression of experimental allergic encephalomyelitis by interferon. Immunol Commun (1982) 11:1–7. doi: 10.3109/08820138209050718 6178681

[15] Paty DW Li DK. Interferon beta-1b is effective in relapsing-remitting multiple sclerosis. II. MRI analysis results of a multicenter, randomized, double-blind, placebo-controlled trial. UBC MS/MRI Study Group and the IFNB Multiple Sclerosis Study Group. Neurology (1993) 43:662–7. doi: 10.1212/wnl.43.4.662 8469319

[16] Yednock TA Cannon C Fritz LC Sanchez-Madrid F Steinman L Karin N. Prevention of experimental autoimmune encephalomyelitis by antibodies against alpha 4 beta 1 integrin. Nature (1992) 356:63–6. doi: 10.1038/356063a0 1538783

[17] Dendrou CA Fugger L Friese MA. Immunopathology of multiple sclerosis. Nat Rev Immunol (2015) 15:545–58. doi: 10.1038/nri3871 26250739

[18] Becher B Segal BM. T(H)17 cytokines in autoimmune neuro-inflammation. Curr Opin Immunol (2011) 23:707–12. doi: 10.1016/j.coi.2011.08.005 PMC3535446 21907555

[19] Kebir H Ifergan I Alvarez JI Bernard M Poirier J Arbour N. Preferential recruitment of interferon-gamma-expressing TH17 cells in multiple sclerosis. Ann Neurol (2009) 66:390–402. doi: 10.1002/ana.21748 19810097

[20] Edwards LJ Robins RA Constantinescu CS. Th17/Th1 phenotype in demyelinating disease. Cytokine (2010) 50:19–23. doi: 10.1016/j.cyto.2009.12.003 20045653

[21] Ajami B Bennett JL Krieger C McNagny KM Rossi FM. Infiltrating monocytes trigger EAE progression, but do not contribute to the resident microglia pool. Nat Neurosci (2011) 14:1142–9. doi: 10.1038/nn.2887 21804537

[22] Moreno MA Burns T Yao P Miers L Pleasure D Soulika AM. Therapeutic depletion of monocyte-derived cells protects from long-term axonal loss in experimental autoimmune encephalomyelitis. J Neuroimmunol (2016) 290:36–46. doi: 10.1016/j.jneuroim.2015.11.004 26711567

[23] Fife BT Huffnagle GB Kuziel WA Karpus WJ. CC chemokine receptor 2 is critical for induction of experimental autoimmune encephalomyelitis. J Exp Med (2000) 192:899–905. doi: 10.1084/jem.192.6.899 10993920 PMC2193286

[24] Izikson L Klein RS Charo IF Weiner HL Luster AD. Resistance to experimental autoimmune encephalomyelitis in mice lacking the CC chemokine receptor (CCR)2. J Exp Med (2000) 192:1075–80. doi: 10.1084/jem.192.7.1075 PMC2193310 11015448

[25] Gaupp S Pitt D Kuziel WA Cannella B Raine CS. Experimental autoimmune encephalomyelitis (EAE) in CCR2(-/-) mice: susceptibility in multiple strains. Am J Pathol (2003) 162:139–50. doi: 10.1016/S0002-9440(10)63805-9 PMC1851120 12507897

[26] Monaghan KL Zheng W Hu G Wan ECK. Monocytes and monocyte-derived antigen-presenting cells have distinct gene signatures in experimental model of multiple sclerosis. Front Immunol (2019) 10:2779. doi: 10.3389/fimmu.2019.02779 31849962 PMC6889845

[27] Jones MV Nguyen TT Deboy CA Griffin JW Whartenby KA Kerr DA. Behavioral and pathological outcomes in MOG 35-55 experimental autoimmune encephalomyelitis. J Neuroimmunol (2008) 199:83–93. doi: 10.1016/j.jneuroim.2008.05.013 18582952

[28] Wickham H. ggplot2: Elegant Graphics for Data Analysis. Switzerland: Springer International Publishing (2016).

[29] Efron B Hastie T. Computer Age Statistical Inference: Algorithms, Evidence, and Data Science. Cambridge, England: Cambridge University Press (2016).

[30] Holm S. A simple sequentially rejective multiple test procedure. Scandinavian J Stat (1979) 6:65–70.

[31] Zhou H Olsen H So E Merigeon E Rybin D Owens J. A fully recombinant human IgG1 Fc multimer (GL-2045) inhibits complement-mediated cytotoxicity and induces iC3b. Blood Adv (2017) 1:504–15. doi: 10.1182/bloodadvances.2016001917 PMC5728453 29296968

[32] Saligrama N Zhao F Sikora MJ Serratelli WS Fernandes RA Louis DM. Opposing T cell responses in experimental autoimmune encephalomyelitis. Nature (2019) 572:481–7. doi: 10.1038/s41586-019-1467-x PMC7145319 31391585

[33] Wagner CA Roque PJ Mileur TR Liggitt D Goverman JM. Myelin-specific CD8+ T cells exacerbate brain inflammation in CNS autoimmunity. J Clin Invest (2020) 130:203–13. doi: 10.1172/JCI132531 PMC6934187 31573979

[34] Plemel JR Stratton JA Michaels NJ Rawji KS Zhang E Sinha S. Microglia response following acute demyelination is heterogeneous and limits infiltrating macrophage dispersion. Sci Adv (2020) 6:eaay6324. doi: 10.1126/sciadv.aay6324 31998844 PMC6962036

[35] Bennett ML Bennett FC Liddelow SA Ajami B Zamanian JL Fernhoff NB. New tools for studying microglia in the mouse and human CNS. Proc Natl Acad Sci USA (2016) 113:E1738–46. doi: 10.1073/pnas.1525528113 PMC4812770 26884166

[36] Butovsky O Jedrychowski MP Moore CS Cialic R Lanser AJ Gabriely G. Identification of a unique TGF-beta-dependent molecular and functional signature in microglia. Nat Neurosci (2014) 17:131–43. doi: 10.1038/nn.3599 PMC4066672 24316888

[37] Keren-Shaul H Spinrad A Weiner A Matcovitch-Natan O Dvir-Szternfeld R Ulland TK. A unique microglia type associated with restricting development of alzheimer's disease. Cell (2017) 169:1276–1290 e17. doi: 10.1016/j.cell.2017.05.018 28602351

[38] Cardona SM Kim SV Church KA Torres VO Cleary IA Mendiola AS. Role of the fractalkine receptor in CNS autoimmune inflammation: new approach utilizing a mouse model expressing the human CX3CR1(I249/M280) variant. Front Cell Neurosci (2018) 12:365. doi: 10.3389/fncel.2018.00365 30386211 PMC6199958

[39] Bonelli M Savitskaya A Steiner CW Rath E Smolen JS Scheinecker C. Phenotypic and functional analysis of CD4+ CD25- Foxp3+ T cells in patients with systemic lupus erythematosus. J Immunol (2009) 182:1689–95. doi: 10.4049/jimmunol.182.3.1689 19155519

[40] Tang Y Peng LP Qin GX Sun JT Xu LJ Jiang YF. CD4(+)CD25(-)Foxp3(+) T cells play a role in tuberculous hydrothorax rather than Malignant hydrothorax. J Transl Med (2015) 13:268. doi: 10.1186/s12967-015-0618-6 26283421 PMC4539708

[41] Karpus WJ. Cytokines and chemokines in the pathogenesis of experimental autoimmune encephalomyelitis. J Immunol (2020) 204:316–26. doi: 10.4049/jimmunol.1900914 31907274

[42] Ghoreschi K Laurence A Yang XP Tato CM McGeachy MJ Konkel JE. Generation of pathogenic T(H)17 cells in the absence of TGF-beta signalling. Nature (2010) 467:967–71. doi: 10.1038/nature09447 PMC3108066 20962846

[43] Lee Y Awasthi A Yosef N Quintana FJ Xiao S Peters A. Induction and molecular signature of pathogenic TH17 cells. Nat Immunol (2012) 13:991–9. doi: 10.1038/ni.2416 PMC3459594 22961052

[44] O'Connor RA Prendergast CT Sabatos CA Lau CW Leech MD Wraith DC. Cutting edge: Th1 cells facilitate the entry of Th17 cells to the central nervous system during experimental autoimmune encephalomyelitis. J Immunol (2008) 181:3750–4. doi: 10.4049/jimmunol.181.6.3750 PMC2619513 18768826

[45] Miyara M Yoshioka Y Kitoh A Shima T Wing K Niwa A. Functional delineation and differentiation dynamics of human CD4+ T cells expressing the FoxP3 transcription factor. Immunity (2009) 30:899–911. doi: 10.1016/j.immuni.2009.03.019 19464196

[46] Mishra MK Yong VW. Myeloid cells - targets of medication in multiple sclerosis. Nat Rev Neurol (2016) 12:539–51. doi: 10.1038/nrneurol.2016.110 27514287

[47] Huitinga I van Rooijen N de Groot CJ Uitdehaag BM Dijkstra CD. Suppression of experimental allergic encephalomyelitis in Lewis rats after elimination of macrophages. J Exp Med (1990) 172:1025–33. doi: 10.1084/jem.172.4.1025 PMC2188611 2145387

[48] Sosa RA Murphey C Ji N Cardona AE Forsthuber TG. The kinetics of myelin antigen uptake by myeloid cells in the central nervous system during experimental autoimmune encephalomyelitis. J Immunol (2013) 191:5848–57. doi: 10.4049/jimmunol.1300771 PMC3891847 24227784

[49] Prinz M Tay TL Wolf Y Jung S. Microglia: unique and common features with other tissue macrophages. Acta Neuropathol (2014) 128:319–31. doi: 10.1007/s00401-014-1267-1 24652058

[50] Wolf Y Shemer A Levy-Efrati L Gross M Kim JS Engel A. Microglial MHC class II is dispensable for experimental autoimmune encephalomyelitis and cuprizone-induced demyelination. Eur J Immunol (2018) 48:1308–18. doi: 10.1002/eji.201847540 29697861

[51] Mundt S Mrdjen D Utz SG Greter M Schreiner B Becher B. Conventional DCs sample and present myelin antigens in the healthy CNS and allow parenchymal T cell entry to initiate neuroinflammation. Sci Immunol (2019) 4. doi: 10.1126/sciimmunol.aau8380 30679199

[52] Garcia JA Pino PA Mizutani M Cardona SM Charo IF Ransohoff RM. Regulation of adaptive immunity by the fractalkine receptor during autoimmune inflammation. J Immunol (2013) 191:1063–72. doi: 10.4049/jimmunol.1300040 PMC3720756 23817416

[53] Fuller JP Stavenhagen JB Teeling JL. New roles for Fc receptors in neurodegeneration-the impact on Immunotherapy for Alzheimer's Disease. Front Neurosci (2014) 8:235. doi: 10.3389/fnins.2014.00235 25191216 PMC4139653

[54] Muller M Carter SL Hofer MJ Manders P Getts DR Getts MT. CXCR3 signaling reduces the severity of experimental autoimmune encephalomyelitis by controlling the parenchymal distribution of effector and regulatory T cells in the central nervous system. J Immunol (2007) 179:2774–86. doi: 10.4049/jimmunol.179.5.2774 17709491

