# Supplementary material for: Fc multimers effectively treat murine models of multiple sclerosis
Source: Front Immunol. 2023 Aug 11;14:1199747. doi: 10.3389/fimmu.2023.1199747 (PMC10451071; doi:10.3389/fimmu.2023.1199747)
Supplement: Supplementary file 1 [file DataSheet_1.pdf]

Supplemental Figures

Supplemental Table 1. M019 inhibits demyelination and inflammation in brains of MOG-immunized B6

Group	Specimen ID	Peak Score	End Score	Inflammation	Demyelination
EAE	9746	1.5	1	Sever with rare Eos, more macs? multifocal	Present, multifocal
	9748	3.5	2	Moderate Multifocal	Present multifocal
	9750	4	2	Very severe with increased cell density and neutrophils.	Present, focal
	9752	4	4.5	Moderate, mostly macs, multifocal	Present, multifocal
	9753	3.5	1	Mild, mostly macs, multifocal	Present, multifocal
	9755	4	2	Mild multifocal	Present, multifocal
EAE+0.2mg M019	9756	3.5	2	Mild, focal	Present, focal
	9758	3	3	Moderate, multifocal	Present, multifocal
	9759	0	0	Absent	Absent
	9760	0	0	Absent	Absent
	9766	0	0	Absent	Absent
	9767	0	0	Absent	Absent
	9768	0	0	Absent	Absent
	9769	0	0	Absent	Absent
	9770	3.5	2	Mild, multifocal	Present multifocal
EAE+2.0mg M019	9771	0	0	Moderate, focal	Present, focal
	9772	0	0	Absent	Absent
	9796	0	0	Absent	Absent
	9774	3.5	0.5	Mild, multifocal	Present, multifocal
	9775	0	0	Absent	Absent
	9776	0	0	Absent	Absent
	9777	1.5	1	Mild, focal	Present, unifocal
	9778	0	0		
	9779	0	0	Absent	Absent
	9780	0	0	Absent	Absent

Supplemental Table 2. primer sequences for cytokines / chemokines measured by qRT-PCR

Target	Primer sequence
GAPDH	Sense 5'-AATGTGTCCGTCGTGGATCT-3'<br>Anti-sense 5'-CCCAGCTCTCCCCATACATA-3'
CCL7 (MCP-3)	Sense 5'-TGGGAAGCTGTTATCTTCAAGACA-3'<br>Anti-sense 5'-CTCGACCCACTTCTGATGGG-3'
CXCL10 (IP-10)	Sense 5'-TGAAAGCGTTTAGCCAAAAAAGG-3'<br>Anti-sense 5'-AGGGGAGTGATGGAGAGAGG-3'
IL-6	Sense 5'-ATGGATGCTACCAAACTGGAT-3'<br>Anti-sense 5'-CCAGGTAGCTATGGTACTCCAGA-3'
IL-18	Sense 5'-CAGGCCTGACATCTTCTGCAA-3'<br>Anti-sense 5'-CTGACATGGCAGCCATTGT-3'
IFN-γ	Sense 5'-GGAGGAACTGGCAAAAGGAT-3'<br>Anti-sense 5'-TTCAAGACTTCAAAGAGTCTGAGG-3'
IL-10	Sense 5'-CAGAGCCACATGCTCCTAGA-3'<br>Anti-sense 5'-GTCCAGCTGGTCCTTTGTTT-3'

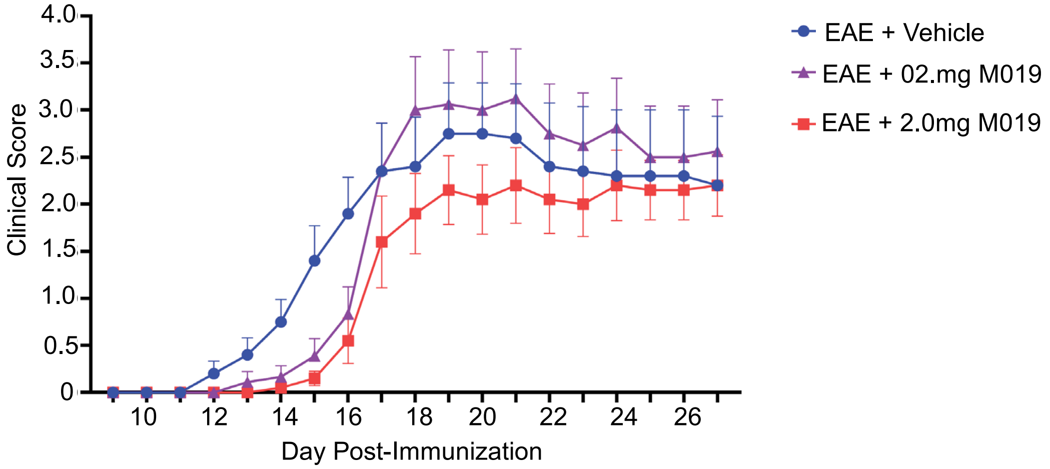

<u>Supplemental Figure 1. M019 treatment on a biweekly basis reduces Disease severity</u>. B6 mice immunized with MOG in CFA emulsion and PTX were treated twice weekly with vehicle, 0.2 mg M019 or 2.0 mg M019 (n=9-10/group). Clinical scores were monitored daily. For mice that reached euthanasia criteria the end clinical score was used for each day following euthanasia. In the EAE+vehicle group 5/10 mice euthanized; EAE+0.2 mg M019 - 3/9 mice euthanized; EAE+2.0mg M019 - 0/10 mice euthanized.

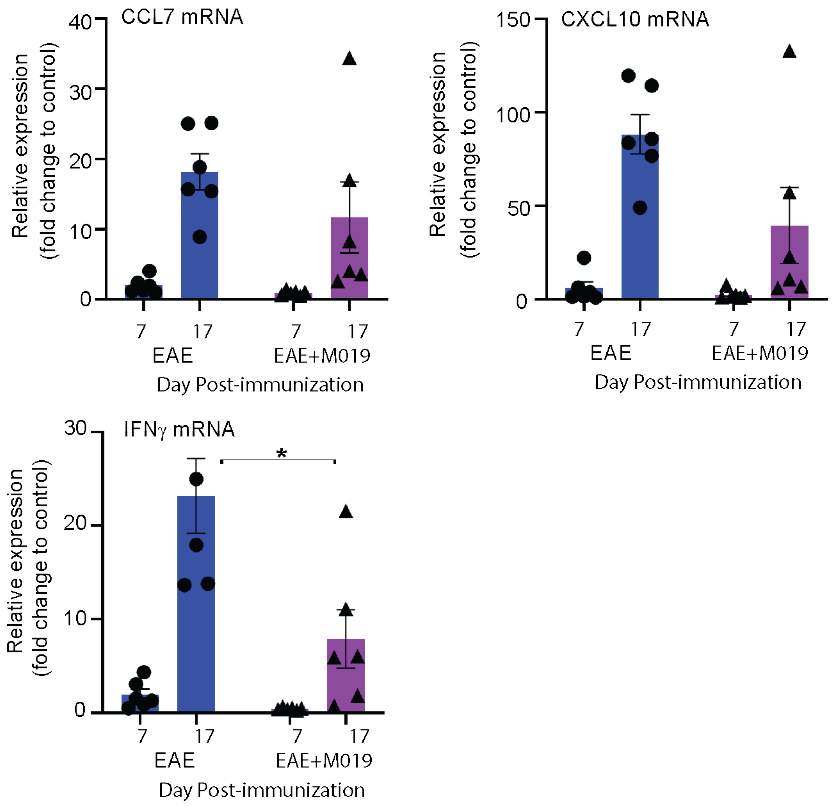

Supplemental Figure 2. Cytokine and chemokine mRNA expression in brains of M019 and vehicle treated EAE induced mice. Relative expression of cytokine and chemokine mRNA in the brain from M019 or vehicle treated mice normalized to the housekeeping gene HPRT and expressed as fold induction over control mice. All panels represent data pooled from 2 experiments with a total n=6 / group; *$p \leq 0.05$, Data analyzed using Mann-Whitney test.
